# Similarities and singularities between innate-like T cell subsets in adults with type 1 diabetes

**DOI:** 10.1016/j.isci.2026.116957

**Published:** 2026-07-27

**Authors:** Isabelle Nel, Gaëlle Vermillac, Léo Bertrand, Lucie Beaudoin, Etienne Larger, Ioannis Theodorou, Agnès Lehuen

**Affiliations:** 1 Université Paris Cité, CNRS, INSERM, Institut Cochin, Paris 75014, France; 2 Immunology Department, AP-HP, Robert-Debré Hospital, Paris 75019, France; 3 Diabetology Department, AP-HP, Cochin Hospital, Paris 75014, France

**Keywords:** autoimmunity, γδT cells, human, iNKT cells, innate-like T cells, MAIT cells, type 1 diabetes

## Abstract

Type 1 diabetes (T1D) is an autoimmune disease for which mouse models have highlighted a role for innate-like T cells, although evidence in humans remains limited. Here, we integrated for the first time flow cytometry analyses of invariant natural killer T (iNKT), γδT, and mucosal-associated invariant T (MAIT) cells from the same adults with recent-onset T1D (*n* = 21), long-term T1D (*n* = 47), and healthy controls (*n* = 55). Recent-onset T1D was associated with reduced frequencies of TNFα-producing iNKT and γδT cells, whereas long-term T1D showed more pronounced alterations, including increased PD-1 expression and an exhaustion-like phenotype across all three populations. Principal-component analysis integrating iNKT, γδT, and MAIT cell parameters positioned recent-onset patients between healthy controls and long-term T1D patients. Exploratory predictive modeling suggests that MAIT cell markers may contribute to disease classification. Together, these findings provide an integrated view of innate-like T cell alterations in adult T1D and highlight their relationship with disease duration.

## Highlights

• Innate like-T cells display a common impaired Th1 profile at T1D onset in adults
• Alterations are exacerbated in long-term patients with an exhaustion-like profile
• iNKT, γδT, and MAIT cell phenotypes also present some unique singularities
• Innate-like T cell features represent potential immune-biomarkers in T1D

## Graphical abstract

**Figure:**
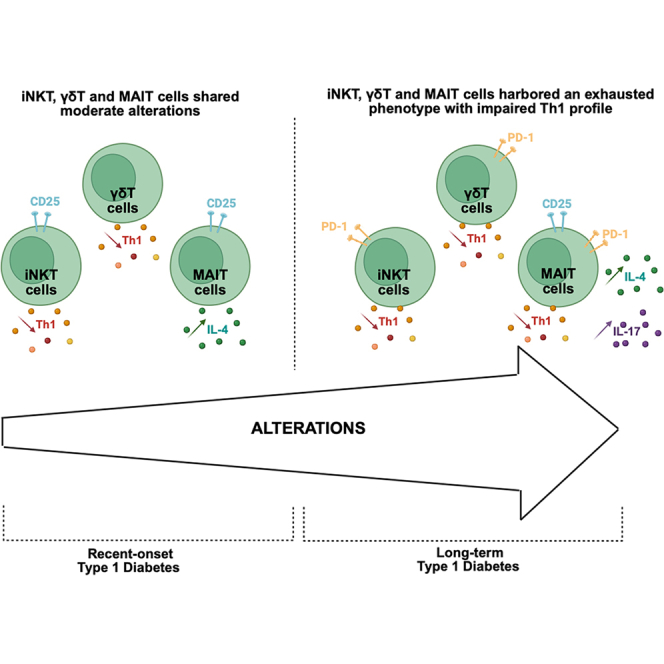

## Introduction

Type 1 diabetes (T1D) is a multifactorial autoimmune disease involving both the innate and adaptive immune systems.^1^^,^^2^^,^^3^ The resulting destruction of the insulin-secreting pancreatic β cells leads to chronic hyperglycemia and a need for life-long insulin-replacement therapy. The challenge of maintaining optimal glycemic control throughout life exposes patients to long-term micro- and macrovascular complications. While most patients develop T1D during childhood, adults represent the majority of patients affected by T1D.^1^^,^^4^^,^^5^

Innate-like T cells, including invariant natural killer T (iNKT), mucosal-associated invariant T (MAIT), and γδT cells, lie at the crossroad between innate and adaptive immunity. These unconventional T cells are characterized either by a restricted γδ or αβ (for iNKT and MAIT cells) T cell receptor (TCR) repertoire. γδT and MAIT cell frequencies in human blood are similar, respectively representing 1%–5% and 1%–10% of blood T cells. In contrast, iNKT cells account for only 0.001%–0.01% of blood T cells.^6^

Unlike conventional T cells, all these cells exhibit an effector-memory phenotype and can be rapidly activated by non-peptidic antigens or cytokines and are recruited to inflamed sites.^7^^,^^8^^,^^9^^,^^10^ iNKT cell TCRs recognize glycolipids or lipid structures,^11^ whereas γδT cell TCRs bind phospho-antigens or self-molecules expressed under cellular stress conditions.^12^ MAIT cells recognize antigens mainly derived from the microbial riboflavin synthesis pathway.^13^ Moreover, antigen recognition in these cells is not restricted to classical major histocompatibility complexes (MHCs). While iNKT and MAIT cells bind non-classical MHCs, γδT cells can recognize antigens either freely or bound to MHCs or MHC-like molecules. Following activation, these cells produce various cytokines, exert cytotoxic functions, or contribute to immune regulation.^7^^,^^8^^,^^9^^,^^10^

We previously highlighted the compromised phenotype and functions of MAIT cells in mouse models of T1D and in the blood of both children and adults with T1D.^14^^,^^15^^,^^16^ In mice, pancreas-infiltrating MAIT cells display pro-inflammatory and cytotoxic features, whereas gut MAIT cells lose protective Th17 cytokine production.^14^^,^^16^ In patients, blood MAIT cells are cytotoxic in children at T1D onset but display a loss of Th1 cytokine production in adults.^14^^,^^15^^,^^17^ In mouse models, iNKT cells are mostly protective against T1D through IL-4, IL-10 production and tolerogenic cell induction,^18^^,^^19^^,^^20^^,^^21^^,^^22^^,^^23^^,^^24^^,^^25^ although studies on iNKT cells in humans have not provided an extensive characterization of these cells.^26^^,^^27^^,^^28^^,^^29^^,^^30^ Regarding γδT cells, some studies in mouse models suggest a protective role^31^; notably through IL-4 and IL-10 production, whereas others support a pathogenic, IL-17-mediated role.^32^^,^^33^ Alterations in circulating γδT cell frequency and phenotype remain unclear as well,^34^^,^^35^^,^^36^^,^^37^ underscoring a need for further γδT cell characterization in human patients with T1D. Moreover, no previous work has simultaneously profiled all three innate-like T cell subsets within the same human cohort.

In this context, the primary objective of this study was to compare and integrate the phenotypic and functional alterations of iNKT, γδT, and MAIT cells in adult T1D patients at different disease stages (recent-onset [RO] vs. long-term [LT]) in peripheral blood, using a cross-sectional design. We performed an extensive analysis of iNKT and γδT cell populations using flow cytometry data previously obtained to characterize MAIT cells in adult patients, allowing an extensive characterization and comparison of alterations in MAIT, γδT, and iNKT cells in patients with RO or LT T1D. Although mechanistic causality cannot be inferred, this approach aimed to characterize immune signatures potentially reflecting disease progression or chronicity. We further leveraged multivariate and predictive modeling approaches to explore key immune features and unconventional T cell subsets associated with T1D status.

## Results

### Circulating iNKT cell phenotype and function are altered in patients with T1D

**Figure 1:**
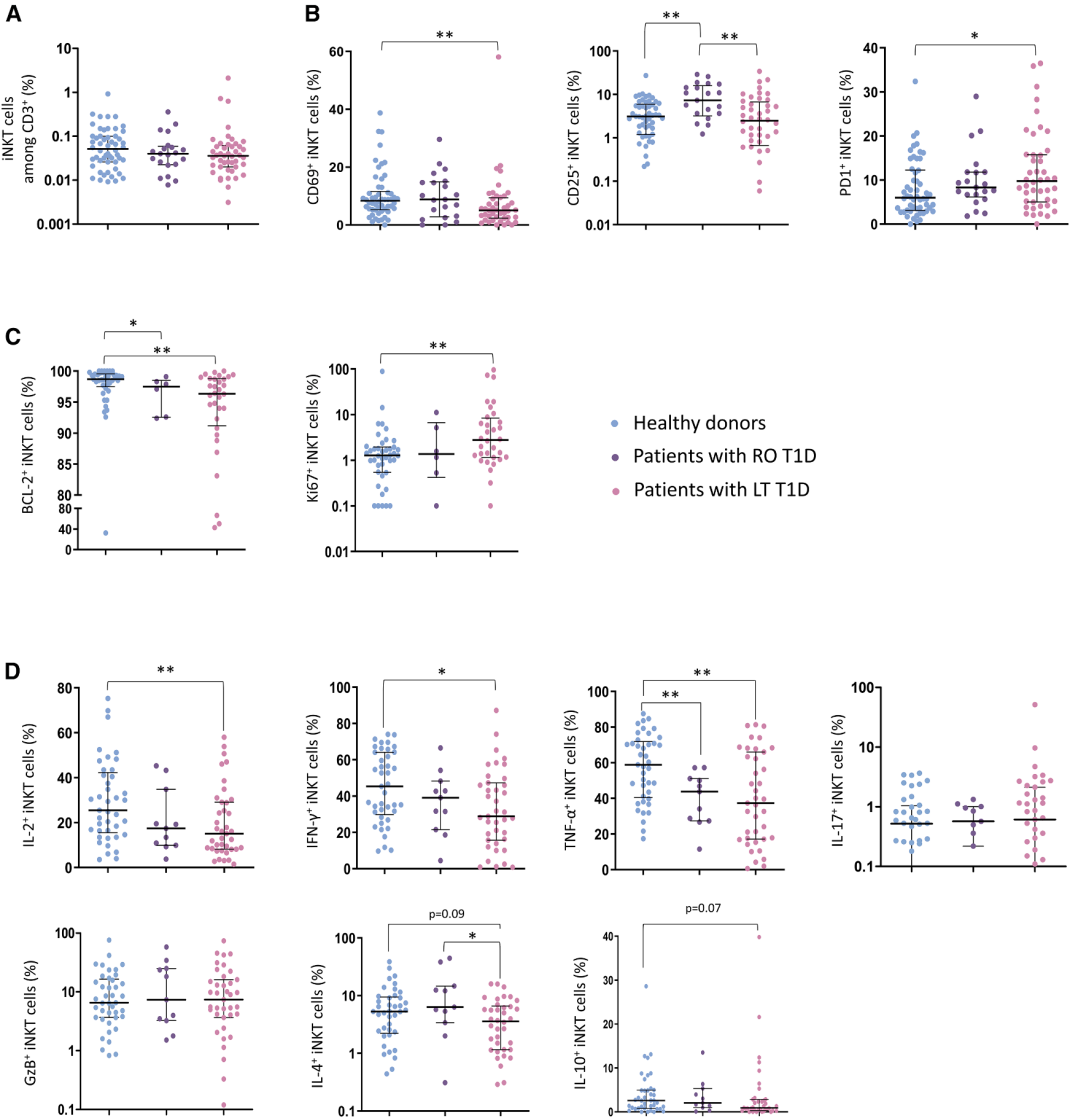
Circulating iNKT cell alterations in adult patients with type 1 diabetes Flow cytometry analyses of blood iNKT cell frequency, phenotype, and function from healthy donors (HDs), recent-onset (RO), or long-term (LT) adult patients with type 1 diabetes (T1D). (A) Circulating iNKT cell frequency (HDs, *n* = 54; RO, *n* = 21; and LT, *n* = 45). (B) Frequencies of CD69^+^, CD25^+^, and PD-1^+^ iNKT cells among total iNKT cells (HDs, *n* = 54; RO, *n* = 21; and LT, *n* = 44). (C) Frequencies of BCL-2^+^ and Ki67^+^ iNKT cells among total iNKT cells (HDs, *n* = 42; RO, *n* = 6; and LT, *n* = 32). For Ki67^+^ iNKT cells, frequencies at 0% were arbitrarily displayed at 0.1% (HDs, *n* = 5; RO, *n* = 1; and LT, *n* = 1). (D) Frequencies of IL-2^+^, IFN-γ^+^, TNF-α^+^, IL-17^+^, granzyme B (GzB)^+^, IL-4^+^, or IL-10^+^ iNKT cells among total iNKT cells (HDs, *n* = 40–41; RO, *n* = 11; and LT, *n* = 37–39). Results from images with limited sample sizes, particularly in RO patients, should be interpreted with caution. Each symbol represents an individual subject, small horizontal lines indicate the median with the interquartile range. ∗*p* < 0.05, ∗∗*p* < 0.01 (non-parametric two-tailed Mann-Whitney test). GzB, granzyme B.

We analyzed, using flow cytometry, the frequency and phenotype of iNKT cells in fresh blood samples from adults with RO T1D and from adults with LT T1D, compared with healthy donors (HDs) (Table S1). iNKT cells were identified based on the expression of the Vα24Jα18 TCR (Figure S1A), and their frequencies were similar among the three groups (Figure 1A). Activation status of iNKT cells was assessed by their expression of CD69, CD25, and PD-1 molecules (Figures 1B and S1B). CD25^+^ iNKT cells were more frequent in patients with RO T1D compared with other groups (*p* = 0.0014 [RO vs. HDs] and *p* = 0.0030 [RO vs. LT]), whereas iNKT cells from patients with LT T1D displayed an exhaustion-like phenotype (increased PD-1 [*p* = 0.029] and decreased CD69 [*p* = 0.0054] expression) (Figure 1B). Accordingly, iNKT cells from this group of patients displayed a decreased expression of the anti-apoptotic B cell lymphoma 2 (BCL-2) molecule compared with healthy adults (*p* = 0.0014). Frequency of Ki67^+^ iNKT cells was also increased in these patients (*p* = 0.0043), suggesting both increased apoptosis and compensatory proliferation in line with their functional exhaustion (Figure 1C). Frequencies of iNKT cells expressing the adhesion molecule CD56, the chemokine receptor CCR6, or the IL-7 receptor CD127 were similar in the three groups (Figures S1B and S2A).

We next evaluated iNKT cell function after PMA-ionomycin stimulation. There was a marked decrease in Th1 cytokine production (IL-2, IFN-γ, and TNF-α) and, to a lesser extent, of IL-4 and IL-10 by iNKT cells from patients with LT T1D (Figures 1D and S1C), consistent with an exhaustion-like phenotype. In patients with RO T1D, only the frequency of TNF-α^+^ iNKT cells was lower than in healthy adults (*p* = 0.0081). However, both granzyme B (GzB)^+^ and IL-17^+^ iNKT cell frequencies were similar among the three groups (Figure 1D). Principal-component analysis (PCA) was performed to assess the distribution of patients and controls according to all observed iNKT cell parameters. The PCA biplot projection of the first and second dimensions showed that patients with RO T1D and those with LT T1D overlapped but were more distinct from healthy adults (Figures S2B and S2C). PERMANOVA analysis revealed a significant global difference between the three groups (R^2^ = 0.056, F = 2.42, and *p* = 0.017). Pairwise comparisons showed that HDs were significantly distinct from LT T1D patients (R^2^ = 0.050, F = 3.76, and *p* = 0.009) and from RO T1D patients (R^2^ = 0.051, F = 2.59, and *p* = 0.025). In contrast, no significant difference was observed between the two T1D groups (*p* = 0.89). Although the PCA biplot projection onto the first and second dimensions reflects only a limited proportion of the total variability (25%), Th1 cytokine production seemed to be the major contributor to the segregation between patients with T1D and HDs.

**Figure 2:**
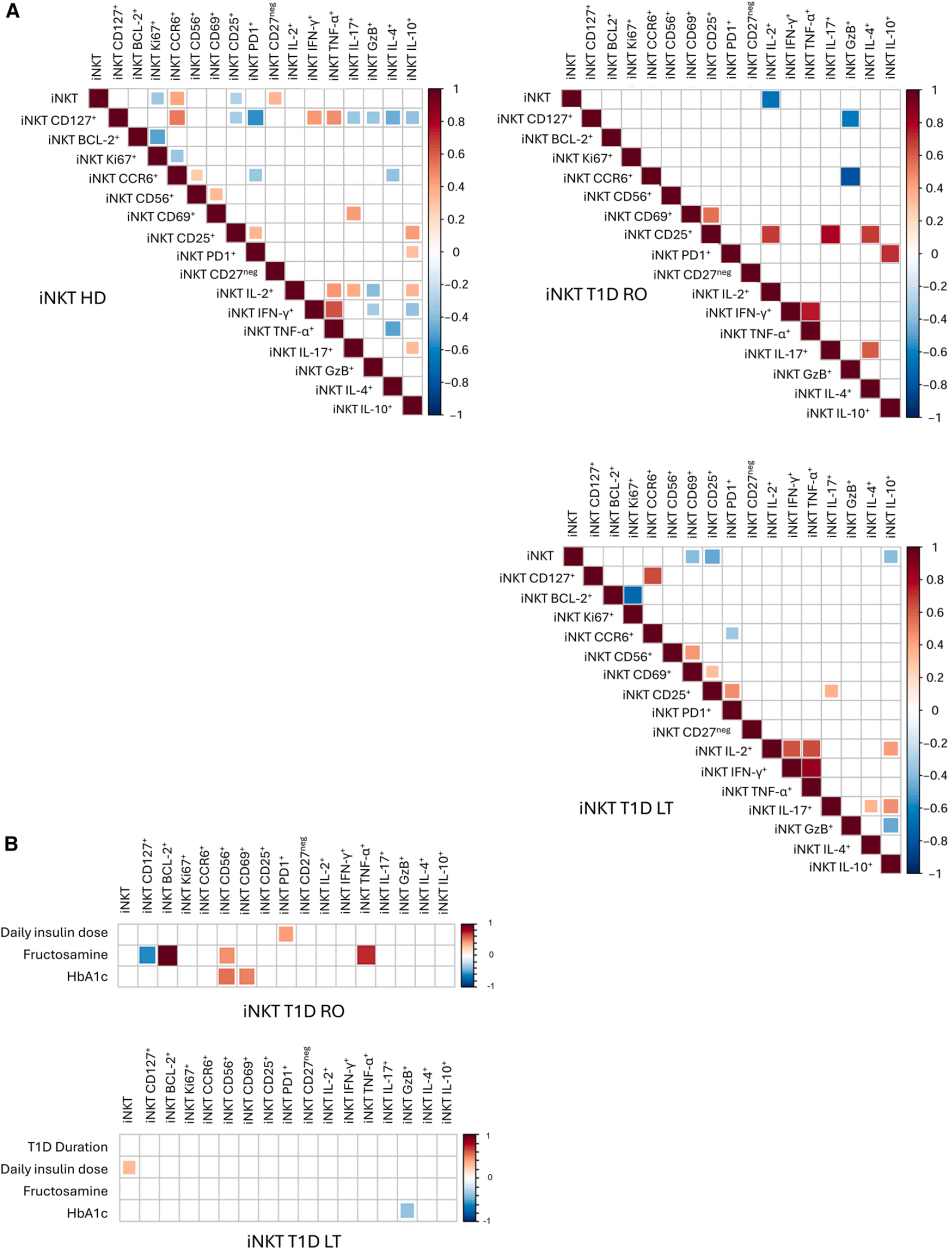
iNKT cell alterations are distinct between patients with recent-onset and long-term type 1 diabetes (A) Correlograms of blood iNKT cell frequency, phenotype, and function in healthy donors (HDs, *n* = 40–54), adult patients with recent-onset (RO, *n* = 6–21), or long-term type 1 diabetes (LT, *n* = 32–45). (B) Correlograms of blood iNKT cell frequency, phenotype, and function with daily insulin dose, fructosamines, HbA1c levels and type 1 diabetes duration in patients with RO type 1 diabetes (*n* = 6–21) or with LT type 1 diabetes (*n* = 32–45). Data were analyzed using Spearman’s non-parametric correlation test. Only statistically significant Spearman correlations (*p* < 0.05) are displayed. Colored squares indicate significant correlations, whereas empty squares correspond to non-significant associations. Color intensity reflects the magnitude and direction of Spearman’s correlation coefficient (ρ), whereas square size is inversely proportional to the *p* value, with larger squares indicating lower *p* values. GzB, granzyme B.

We next sought to identify correlations between all studied iNKT cell parameters. Correlograms of all iNKT cell parameters showed that most correlations observed in HDs were lost in both groups of patients with T1D (Figure 2A). In patients with RO T1D, we observed strong correlation coefficients despite a small number of patients, especially between CD25 and IL-2 and IL-17 (Figure S3), supporting the association between iNKT cell activation and cytokine production in patients with RO T1D. Interestingly, in patients with LT T1D, the correlation between CD25^+^ and IL-2^+^ iNKT cells was lost compared with patients with RO T1D. Moreover, the production of the three Th1 cytokines (IL-2, IFN-γ, and TNF-α) was positively correlated with each other, suggesting that a decrease in these Th1 cytokines occurs in the same patients (Figure 2A).

We next investigated whether iNKT cell frequency, phenotype, and functions were associated with diabetes-related clinical variables (Figure 2B). In patients with RO T1D, CD56^+^ iNKT cell frequency positively correlated with HbA1c and fructosamine levels, the latter being correlated with TNF-α^+^ iNKT cell frequency as well. Moreover, CD69^+^ and PD-1^+^ iNKT cell frequencies positively correlated with HbA1c levels and daily insulin dose, respectively, suggesting an overall association between iNKT cell activation and worsened clinical parameters in these patients at onset. In patients with LT disease, GzB^+^ iNKT cell frequency was negatively correlated with HbA1c levels and iNKT cell frequency positively correlated with daily insulin dose (Figure 2B). Overall, iNKT cell alterations and activation parameters displayed higher correlations with clinical parameters at T1D onset than during follow-up. However, due to the relatively limited size of the RO T1D group (*n* = 21), these correlation analyses should be interpreted cautiously. While several associations reached statistical significance, the limited sample size may have reduced our ability to detect additional clinically relevant correlations.

### Circulating γδT cell phenotype and function are altered in patients with T1D

**Figure 3:**
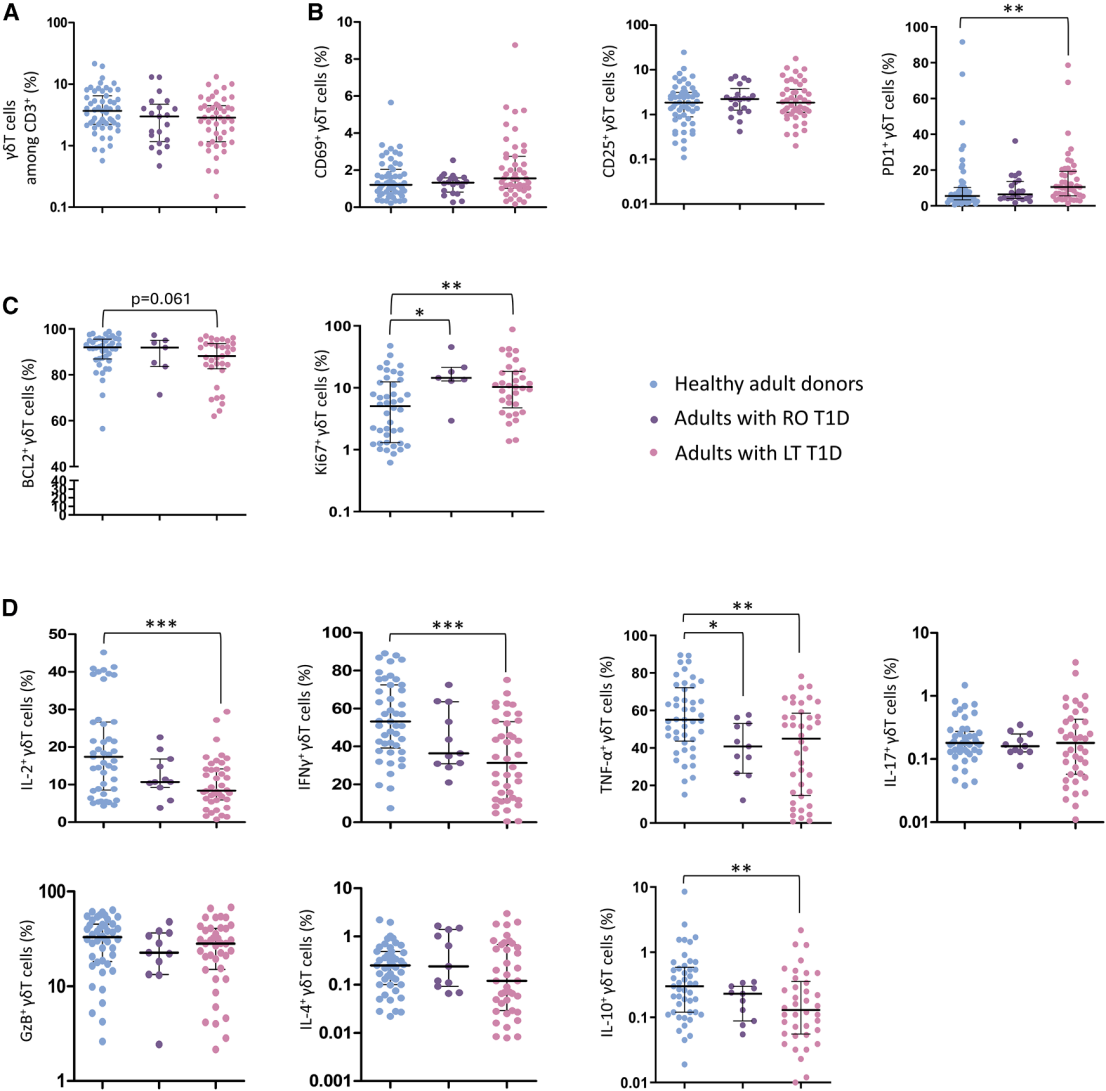
Circulating γδT cell alterations in adult patients with type 1 diabetes Flow cytometry analyses of blood γδT cell frequency, phenotype, and function from HDs, RO, or LT adult patients with type 1 diabetes (T1D). (A) Circulating γδT cell frequency (healthy donors [HDs], *n* = 55; recent-onset [RO], *n* = 21; and long-term [LT], *n* = 47). (B) Frequencies of CD69^+^, CD25^+^, and PD-1^+^ γδT cells among total γδT cells (HDs, *n* = 55; RO, *n* = 21; and LT, *n* = 47). (C) Frequencies of BCL-2^+^ and Ki67^+^ γδT cells among total γδT cells (HDs, *n* = 42; RO, *n* = 7; and LT, *n* = 33). (D) Frequencies of IL-2^+^, IFN-γ^+^, TNF-α^+^, IL-17^+^, granzyme B (GzB)^+^, IL-4^+^, or IL-10^+^ γδT cells among total γδT cells (HDs, *n* = 45; RO, *n* = 11; LT, *n* = 38–40). Results from images with limited sample sizes, particularly in RO patients, should be interpreted with caution. Each symbol represents an individual subject. Small horizontal lines indicate the median with the interquartile range. ∗*p* < 0.05, ∗∗*p* < 0.01, ∗∗∗*p* < 0.001 (non-parametric two-tailed Mann-Whitney test).

We next studied γδT cells by flow cytometry, as performed for iNKT cells. Total γδT cell frequency was similar among all three groups, as were frequencies of γδT cells expressing CCR6, CD56, CD127, CD69, and CD25, indicating no differences in early and late-activation of γδT cells (Figures 3A, 3B, S1A, S4A, and S5A). However, frequencies of PD-1^+^ (*p* = 0.0036) and Ki67^+^ γδT cells (*p* = 0.0048) were increased in patients with LT T1D compared with HDs. BCL-2^+^ γδT cell frequency also tended to decrease in these patients, albeit not significantly (*p* = 0.061) (Figures 3B, 3C, and S4A). Overall, this suggests a similar exhaustion-like profile in γδT cells as in iNKT cells in patients with LT diabetes. Cytokine production analysis showed that in patients with LT T1D, frequencies of all Th1 cytokines and IL-10-producing γδT cells significantly decreased compared with HDs, supporting an exhaustion-like functional profile of these cells as for iNKT cells in these patients (Figures 3D and S4B). In patients with RO T1D, only the frequency of TNF-α^+^ γδT cells decreased compared with HDs, suggesting that this exhaustion-like phenotype is acquired later, after disease onset (*p* = 0.0110). We similarly performed PCA analysis using γδT cell parameters (Figures S5B and S5C). PERMANOVA analysis revealed a significant global difference between the three groups (R^2^ = 0.073, F = 3.55, and *p* = 0.003). Pairwise comparisons showed that HDs were significantly distinct from LT T1D patients (R^2^ = 0.070, F = 5.99, and *p* = 0.001). A trend toward significance was observed between HDs and RO T1D patients (R^2^ = 0.039, F = 2.19, and *p* = 0.082), whereas no significant difference was found between the two T1D groups (*p* = 0.69). The biplot PCA projection representing the first and second principal components suggested that Th1 cytokine-producing γδT cell frequencies may be among the main variables contributing to the segregation axis.

**Figure 4:**
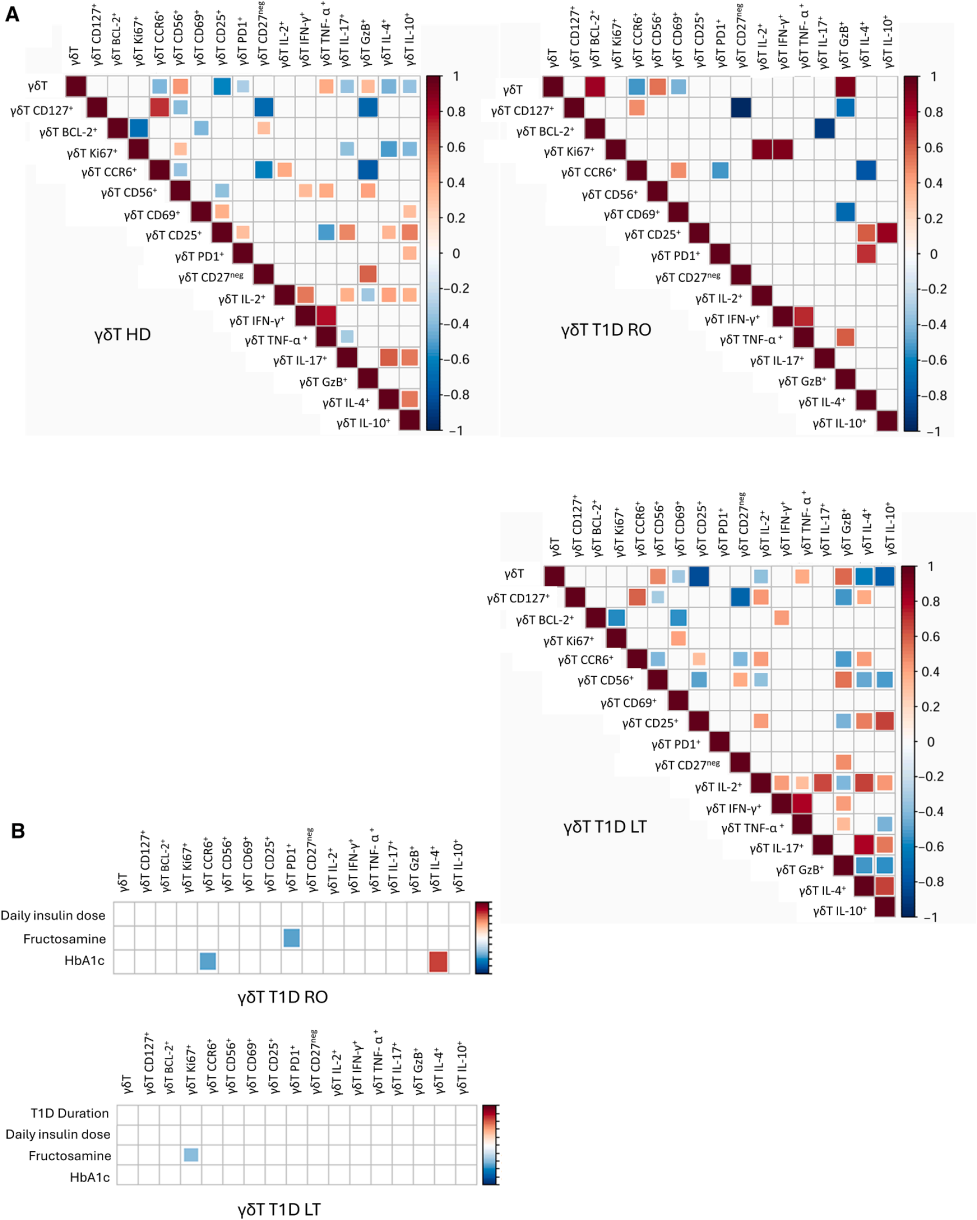
γδT cell alterations are distinct between patients with recent-onset and long-term type 1 diabetes (A) Correlograms of γδT cell frequency, phenotype and function in healthy donors (HDs, *n* = 42–55), adult patients with recent-onset (RO, *n* = 7–21), or long-term (LT, *n* = 38–47) type 1 diabetes. (B) Correlations between γδT cell frequency, phenotype, and function with daily insulin dose, fructosamines or HbA1c levels in patients with RO (*n* = 7–21) type 1 diabetes or in patients with LT (*n* = 38–47) type 1 diabetes. Data were analyzed using Spearman’s non-parametric correlation test. Only statistically significant Spearman correlations (*p* < 0.05) are displayed. Colored squares indicate significant correlations, whereas empty squares correspond to non-significant associations. Color intensity reflects the magnitude and direction of Spearman’s correlation coefficient (ρ), whereas square size is inversely proportional to the *p* value, with larger squares indicating lower *p* values. GzB, granzyme B.

Correlograms of all γδT cell parameters highlighted a stronger degree of correlation similarities between HDs and patients with LT T1D (Figure 4A). We observed a similar correlation of Th1 cytokine production parameters with each other (IL-2, IFN-γ, and TNF-α) as for iNKT cells (Figure 2A). Conversely, patients with RO T1D exhibited fewer correlations, mostly between IL-4, IL-10 production and CD25, and PD-1 expression by γδT cells (Figure S5D). Finally, we observed only a few correlations between γδT cell parameters and clinical data, both in adults with RO and LT T1D (Figure 4B). Our results reveal thus an exhaustion-like profile of γδT cells with a significant loss of Th1 cytokine production in patients with LT T1D but limited correlations with clinical parameters of the disease.

### Polyfunctional Th1 iNKT and γδT cell populations are reduced in patients with T1D

**Figure 5:**
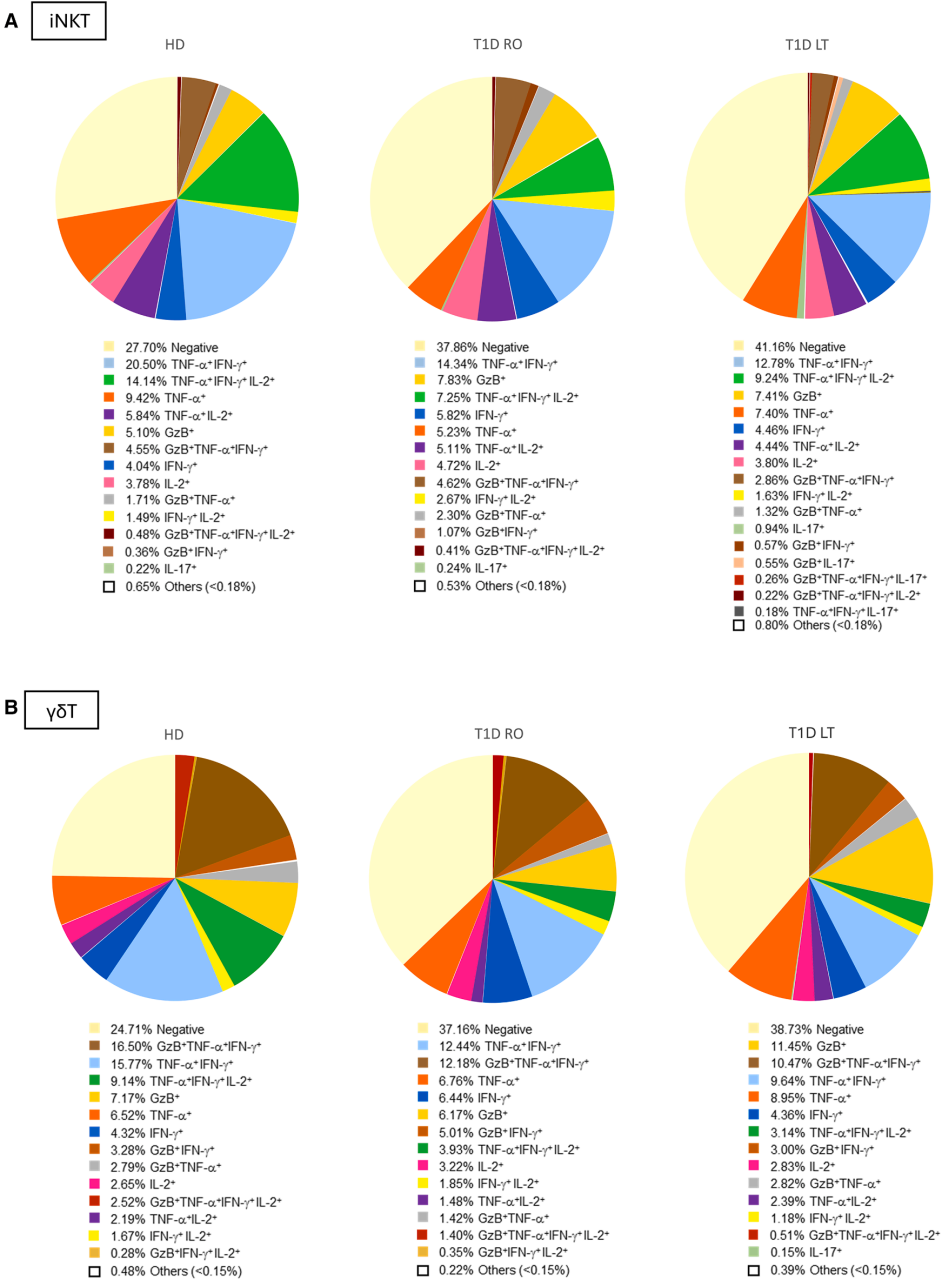
Circulating polyfunctional iNKT and γδT cell subsets are altered in type 1 diabetes (A) Pie charts showing the relative median frequency of iNKT cell functional subsets in healthy donors (HDs, *n* = 40), patients with recent-onset (RO, *n* = 11), or long-term (LT, *n* = 37) type 1 diabetes (T1D). Functional subsets were defined by Boolean analysis of intracellular production of IFN-γ, TNF-α, IL-2, IL-17, and granzyme B (GzB), following stimulation. Pie sectors represent cells producing only a single (monofunctional) or multiple mediators (2, bifunctional; ≥3, polyfunctional cells), whereas the “Negative” fraction corresponds to cells producing none of the measured mediators. Th1 cytokines include IFN-γ, TNF-α, and IL-2. (B) Pie charts showing the relative median frequency of circulating γδT cell functional subsets in HDs (*n* = 45), patients with RO (*n* = 11), or LT (*n* = 38) T1D, defined by Boolean analysis of IFN-γ, TNF-α, IL-2, IL-17, and GzB production. Pie sectors represent mono-, bi-, or polyfunctional subsets, whereas “Negative” indicates cells negative for all measured mediators.

We next performed a polyfunctional analysis to evaluate the relative frequencies of iNKT and γδT cells producing multiple mediators such as Th1 cytokines, GzB, and IL-17 (Figures 5 and S6). Negative and polyfunctional cells were, respectively, defined as cells producing none or at least three of the studied cytokines (IFN-γ, TNF-α, IL-2, IL-17, and GzB). Polyfunctional iNKT and γδT cell frequencies were significantly decreased (*p* = 0.006 for iNKT and *p* < 0.0001 for γδT cells) in patients with LT T1D compared with healthy controls (Figures S6A–S6D). Bifunctional iNKT and γδT cells were also reduced in these patients. Conversely, negative iNKT and γδT cell frequencies were increased in patients with LT disease (*p* = 0.02 for iNKT; *p* = 0.002 for γδT cells). Interestingly, negative and polyfunctional γδT cell frequencies were increased (*p* = 0.001) and decreased (*p* = 0.02), respectively, in patients with RO T1D (Figures S6A–S6D). These population shifts mainly resulted from the reduction of Th1 cytokine production observed in iNKT and γδT cells, as Th1 cytokines are quantitatively the most abundant cytokines produced by these cells (Figures 1D and 3D). Both Th1 and Th1/GzB polyfunctional cells were decreased for both innate-like cell types (Figure 5). Analyses of polyfunctionality also highlighted some specificities of each innate-like cell population. In healthy individuals, the Th1/GzB subset reached 25% of γδT cells compared with 7% of iNKT cells. Conversely, we detected IL-17-producing subsets in iNKT cells only, which both increased in frequency (0.94% in LT vs. 0.22% in HDs for IL-17-only producing iNKT cells) and co-expressed a greater degree of Th1 cytokines and/or GzB in patients with LT T1D (Figure 5).

### Innate-like T cells share mostly common, exacerbated alterations compared with conventional T cells

To provide a comprehensive overview and facilitate comparison of innate-like T cell alterations in patients with T1D against HDs, we summarized the alterations of MAIT cells previously reported^15^ alongside those of iNKT and γδT cells described in this study (Table 1). Those comparisons aimed at highlighting shared or distinct patterns across innate-like T cell subsets. In adults with RO T1D, iNKT cells expressed a late activation profile characterized by CD25 upregulation, a feature also observed in MAIT cells (Figure 1B and Table 1). However, iNKT cells also displayed signs of altered cell homeostasis and reduced Th1 cytokine production, which were also observed in γδT cells (Figures 1B and 3B; Table 1). Therefore, iNKT cells displayed a profile characterized by multiple features also observed in MAIT or γδT cells. Of note, only MAIT cells in these patients produced higher levels of IL-4.

**Table 1:** Differential alterations of innate-like T and conventional T cells in adult patients with RO T1D or with LT T1D

RO T1D (vs. HDs)		Innate-like T cells			Conventional T cells		LT T1D (vs. HDs)		Innate-like T cells			Conventional T cells	
		MAIT	iNKT	γδT cells	CD4 T cells	CD8 T cells			MAIT	iNKT	γδT cells	CD4 T cells	CD8 T cells
Frequency		–	–	–	–	–	Frequency		(↘)	–	–	–	–
Migration Adhesion	CCR6 CD56	–	–	–	–	–	Migration Adhesion	CCR6 CD56	–	–	–	–	–
Activation Exhaustion	CD69	–	–	–	–	–	Activation Exhaustion	CD69	–	↘∗∗	–	↗∗∗∗	–
	CD25	↗∗∗∗	↗∗∗		↗∗∗	↗∗∗		CD25	↗∗∗∗	–	–	–	↗∗∗∗
	PD-1	–	–	–	–	–		PD-1	↗∗∗∗	↗∗	↗∗∗	↗∗	–
Proliferation Apoptosis	CD127	–	–	–	–	–	Proliferation Aptotosis	CD127	↗∗	–	–	–	–
	Ki67	–	–	↗∗	–	–		Ki67	↗∗∗	↗∗∗	↗∗∗	(↗)	(↗)
	BCL-2	–	↘∗		–	–		BCL-2	–	↘∗∗	(↘)	–	–
Cytokines	Th1 (IFNγ, TNF, IL-2)		↘∗∗	↘∗	↘∗	↘∗	Cytokines	Th1 (IFNγ, TNF, IL-2)	↘∗∗	↘∗∗	↘∗∗∗	–	–
	IL-17	–	–	–	–	–		IL-17	↗∗	–	–	–	–
	IL-4	↗∗∗	–	–	–	–		IL-4	↗∗∗	–	–	–	–
	IL-10	–	–	–	–	–		IL-10	–	(↘)	↘∗∗	–	–
Cytotoxicity	GzB	–	–	–	–	↘∗	Cytotoxicity	GzB	–	–	–	–	–

Alterations of innate-like and conventional T cell parameters in patients with recent-onset (RO) or long-term (LT) type 1 diabetes, compared with healthy donors (HDs). Data are presented as arrows and stars representing significant *p* values (∗*p* < 0.05, ∗∗*p* < 0.01, ∗∗∗*p* < 0.001, non-parametric two-tailed Mann-Whitney test). Upward arrows indicate an increase in the frequency of MAIT, iNKT, γδT, and/or conventional T cells expressing the specified marker or cytokine. Downward arrows indicate a decrease in the frequency of MAIT, iNKT, γδ T cells, and/or conventional T cells expressing the specified marker/cytokine. Arrows in brackets represent a trend toward an increase or decrease without reaching statistical significance. GzB, granzyme B.

In adults with LT T1D, the three innate-like T cell populations displayed an exhaustion-like profile with PD-1 upregulation associated with impaired Th1 cytokine production and increased proliferation (Figures 1B and 3B; Table 1). Both iNKT and γδT cells produced low levels of IL-4 and IL-10, whereas MAIT cells produced increased levels of IL-4 and IL-17. Overall, innate-like T cells showed overlapping features of an altered phenotype in adults with LT T1D, suggesting a partially convergent pattern of immune dysregulation across all three subsets. We also analyzed the same flow cytometry parameters in conventional CD4 and CD8 T cells (Figure S7) and compared their alterations with those of innate-like T cells (Table 1). In patients with RO T1D, frequencies of both CD25^+^ CD4 (*p* = 0.0086) and CD25^+^ CD8 T cells (*p* = 0.0019) were increased as observed for MAIT and iNKT cells (Figures S7A and S7C). Th1 cytokine production was reduced in conventional T cells, though to a lesser extent compared to iNKT and γδT cells (Figures S7B and S7D; Table 1). Regarding patients with LT T1D, CD25^+^ CD8 T cell frequency was increased (*p* = 0.0007), as observed for MAIT cells (Figure S7C; Table 1). CD69^+^ CD4 T cell frequency was increased (*p* = 0.0002), a feature not observed in any other T cell populations. Altogether, in patients with T1D, innate-like T cells showed more marked phenotypic alterations than conventional T cells.

### Relative importance of MAIT, iNKT, and γδT cells parameters to predict T1D status

**Figure 6:**
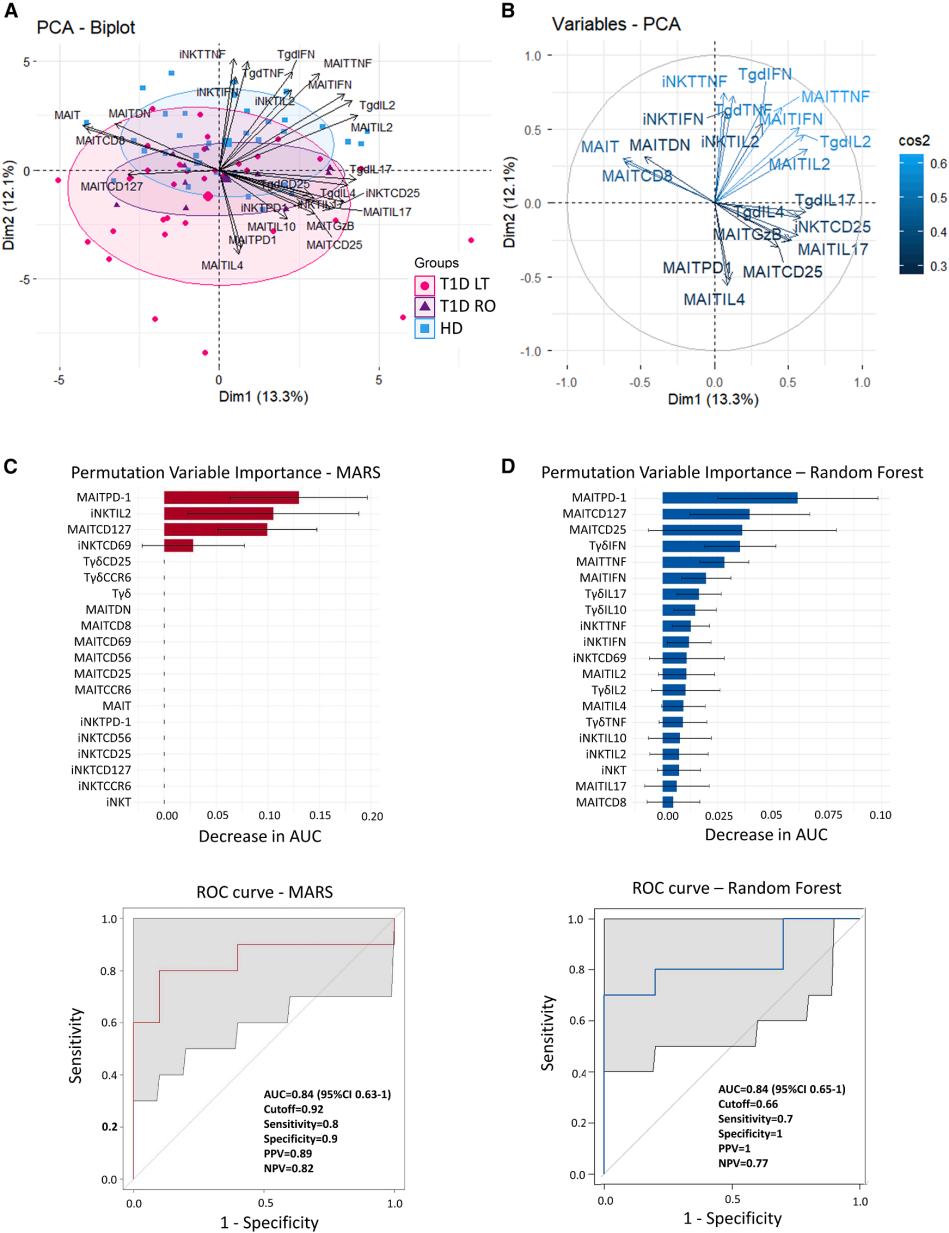
Innate-like T cells parameters in type 1 diabetes status prediction (A and B) Principal-component analysis (PCA) of healthy donors (HDs, *n* = 34), recent-onset (RO, *n* = 10), and long-term (LT, *n* = 34) adult patients with type 1 diabetes, using MAIT cells, iNKT cells, and γδT cells frequency, phenotype, and function markers as variables. Biplot PCA projection represents the first and second dimensions. Arrows represent the contributions of each quantitative variable. (A) Each small point represents a single patient, and concentration ellipses indicate 95% confidence intervals for each group. (B) Arrows are colored based on the square cosine, which is representative of variable fit on the dimensions. (C and D) MARS (C) and random (D) predictive models output of major innate-like T cell variables discriminative for type 1 diabetes status between HDs (*n* = 34) and LT (*n* = 34) adult patients with type 1 diabetes. Input for each model included MAIT cell, iNKT cell, γδT cell frequency, phenotype, and function markers as variables. For each predictive model, upper image shows the results of the variable permutation analysis, highlighting the most contributory variables. Lower image corresponds to the associated receiver-operating characteristic (ROC) curve. AUC, area under curve; PPV, positive predictive value; NPV, negative predictive value.

We next investigated the relative importance of innate-like T cell parameters predicting clinical status. PCA analysis and predictive models were therefore applied only to patients and controls without missing values, resulting in the inclusion of 34 adults with LT T1D, 10 adults with RO T1D, and 34 healthy controls. PCA was used to analyze the distribution of patients and/or controls based on MAIT, iNKT and γδT cell frequencies, phenotype, and functional parameters. PERMANOVA analysis revealed a significant global difference between the three groups (R^2^ = 0.077, F = 3.20, and *p* = 0.002). Pairwise comparisons showed that HDs were significantly distinct from LT T1D patients (R^2^ = 0.072, F = 5.30, and *p* = 0.001) and from RO T1D patients (R^2^ = 0.051, F = 2.36, and *p* = 0.014). In contrast, no significant difference was observed between the two T1D groups (*p* = 0.56). The PCA biplot (Figures 6A and 6B) representing the first two principal components, which accounted for 25% of the total variance, suggested that patients with RO T1D clustered intermediate between healthy adult controls and adults with LT T1D. The variables contributing most strongly to this distribution were primarily MAIT cell-related (PD-1 and IL-4 expression) together with Th1 cytokine production (IL-2, IFN-γ, and TNF-α) across all innate-like T cell subsets.

We next executed on our dataset two different predictive models to extract parameters most associated with disease status: one based on ensemble learning (random forest), the other on linear spline regression (MARS). Given the small number of patients with RO T1D, we first analyzed data by grouping patients with LT T1D and patients with RO T1D together under the diabetes phenotype. However, these combined analyses were deemed insufficiently reliable with limited to moderate discriminative performance (Figures S8A–S8D).

We next performed predictive models restricted to patients with LT T1D and healthy controls (Figures 6C and 6D). As expected, the linear model retained the most significant variables among correlated ones. Both models suggested that MAIT cells may be among the variables contributing most to the classification between patients with LT T1D and healthy controls, and particularly PD-1^+^ MAIT cells and CD127^+^ MAIT cells. Despite the limitations related to the small number of patients, both models yielded area under curve (AUC) values of approximately 0.84, suggesting a moderate-to-good discriminatory performance, even though the wide amplitude of the AUC confidence interval highlights the exploratory nature of these analyses. Moreover, the sensitivity values obtained at the optimal threshold were relatively modest for both models, indicating that while MAIT cells appear to be among the variables contributing most to the classification between LT T1D patients and HDs, their individual discriminatory power remains limited. These results should therefore be interpreted with caution, only with an exploratory approach and will require validation in larger independent cohorts.

## Discussion

This study provides a detailed phenotypic and functional characterization of innate-like T cells in peripheral blood from adult patients with T1D. In this study, we observed in patients with RO T1D an impaired Th1 cytokine production in iNKT and γδT cells, a defect that is more pronounced in patients with LT T1D. In these patients, iNKT and γδT cells displayed an exhaustion-like phenotype and a reduced production of IL-4 and IL-10. Several of these alterations correlated with clinical parameters. MAIT cells from the same patients^15^ displayed similar alterations to those observed for iNKT and γδT cells, with some distinct features. PCA and predictive models, performed with all three innate-like T cell populations, suggested the relative importance of MAIT cell parameters and Th1 cytokine production to differentiate patients with T1D from HDs.

Although there is no consensus on iNKT cell frequency in adults with T1D, our findings align with recent reports.^28^^,^^30^ Regarding cell surface molecules, both CD56 and CD69 expression on iNKT cells positively correlated with HbA1c levels in patients with RO T1D. Although the functional impact of CD56^+^ iNKT cells is not well defined, CD56 expression on MAIT cells is associated with higher responsiveness to cytokine stimulation^38^ and higher deleterious cytotoxic function.^39^ Previous studies have highlighted that activated iNKT cells promote IFN-α production by plasmacytoid dendritic cells,^25^ which have an increased ability to exert antigenic presentation in adults with T1D.^40^ A potential direct impact of activated iNKT cells on pancreatic β cells, or indirect through macrophages and dendritic cells, remains to be further investigated.

We identified a small subset of IL-17-producing iNKT cells across all three groups of adults. In adults with LT T1D, IL-17 production was associated with Th1 cytokine and GzB production, similar to what was observed for MAIT cells in the same cohort.^15^ Although IL-17^+^ iNKT cell frequency did not correlate with clinical parameters, these cells may promote islet destruction as described in non-obese diabetic (NOD) mice.^23^ However, no correlation was found between activation and IL-17 production in γδT cells and the involvement of IL-17^+^ γδT cells in T1D still remains unclear.^33^^,^^41^ Altered production of immunoregulatory (IL-10) and Th2 cytokines (IL-4 and IL-13) by iNKT cells in adults with T1D has been previously reported,^26^^,^^28^^,^^30^ and the key role of immunoregulatory iNKT cells against T1D demonstrated in NOD mice.^19^^,^^21^^,^^22^^,^^24^ iNKT cells exert several protective mechanisms, including production of regulatory cytokines and induction of tolerogenic dendritic cells, anergic anti-islet T cells and regulatory T cells in pancreatic lymph nodes.^21^^,^^22^^,^^24^ Although our analysis of peripheral blood mononucleated cells (PBMCs) did not allow to investigate such mechanisms, we confirmed a decrease of IL-4^+^ iNKT cells and showed a reduction of IL-10^+^ γδT cells in adults with LT T1D. iNKT cells and γδT cells tend to have a protective role in T1D by their production of IL-4 and IL-10,^18^^,^^19^^,^^20^^,^^21^^,^^22^^,^^24^^,^^26^^,^^28^^,^^31^ while a pathogenic role of MAIT cells is often associated with their production of cytotoxic molecules.^14^^,^^39^^,^^42^ Thus, the impaired function of iNKT and γδT cells might be associated with a loss of IL-4 and IL-10, whereas the chronic inflammation might induce IL-4 production by MAIT cells.^15^^,^^43^ Taken together, these observations are consistent with an alteration of innate-like T cell function due to chronic activation and inflammation, inducing loss of a regulatory role of iNKT and γδT cells, and a shift of MAIT cells toward a weak Th2/Th17 phenotype at later stages of the disease in adults.

Conversely, all innate-like T cells exhibited significant impaired Th1 cytokine production and PD-1 upregulation in adults with LT T1D, features consistent with functional exhaustion of these cells. While we focused here on PD-1 as a key marker of dysfunction, future work could include additional exhaustion-associated markers (e.g., TIM-3 and LAG-3) or single-cell RNA sequencing to better characterize this exhaustion-like profile. Conversely, a partial loss of Th1 cytokine production was observed in iNKT and γδT cells in adults with RO T1D. Accordingly, the increase in negative and conversely decrease in polyfunctional cell frequencies in patients with LT diabetes was also observed for γδT cells in patients with RO T1D. A similar phenotype was also observed for MAIT cells, and at a greater level in patients with LT T1D than in RO patients.^15^ Therefore, loss of Th1 cytokine production is shared across all innate-like T cells and is more pronounced in patients with longer disease duration. Patients included in our cohort had mostly well-controlled blood glucose levels and no major complications, excluding poor glycemic control as a cause for this degradation. This exhausted-like phenotype may rather result from prolonged cytokine-mediated inflammation, which is sufficient to activate innate-like T cells through TCR-independent pathways. Indeed, levels of pro-inflammatory blood cytokines (IL-1β, IL-6, TNF, and IL-18) remain elevated in T1D patients long after disease onset,^44^^,^^45^^,^^46^ suggesting ongoing inflammation unresolved by insulin replacement therapy. Interestingly, innate-like T cells are distinct from conventional T cells, whose Th1 cytokine production was altered in RO patients only and normal in patients with long-term disease. Innate-like T cells, which are mostly effector/memory cells, are likely more sensitive to long-term elevated cytokine levels due to higher expression of their receptors.^39^^,^^47^ Such functional impairment may dampen the ability of innate-like T cells to efficiently orchestrate antimicrobial immune responses, given their critical role in priming other immune cells and controlling bacterial and viral infections.^7^^,^^8^^,^^9^^,^^10^

PCA analysis and the two predictive models suggested MAIT cell parameters as contributing variables to predict clinical status. Due to the low number of adults with RO T1D, predictive models were run on both HDs and merged adults with T1D, but with insufficiently reliable results. We next performed predictive models restricted to patients with LT T1D and healthy controls. Both models suggested that MAIT cells may be among the variables contributing the most to the classification, despite the wide amplitude of the AUC confidence interval, highlighting the exploratory nature of these analyses. Nevertheless, for both models, PD-1^+^ MAIT cells and CD127^+^ MAIT cells parameters were highlighted. Interestingly, PD-1^+^ MAIT cells appeared also as main contributory marker in PCA and correlated with HbA1c levels in adults with LT T1D.^15^ In children with T1D, surface MAIT cell parameters were sufficient to perform receiver-operating characteristic discrimination between healthy children and children with T1D.^14^ Altogether, these results suggest some MAIT cell parameters may be used as general biomarkers of disease.

Increasing evidence suggests MAIT cell alterations similar to those reported in T1D have been reported in patients with other autoimmune diseases such as arthritis, lupus, or autoimmune hepatitis.^8^^,^^48^ Moreover, patients with T1D are at high-risk to develop additional auto-immune diseases.^49^ We previously described increased alterations of MAIT cells in women with long-term diabetes affected by a second autoimmune disease, compared with women affected with LT T1D only.^15^ The shared alteration profile observed across innate-like T cells in patients with T1D may represent a broader feature at least partially found in other autoimmune and chronic inflammation diseases. Similar alterations of iNKT and γδT cell have also been reported in multiple sclerosis, psoriasis, lupus, inflammatory bowel diseases, primary biliary cirrhosis or rheumatoid arthritis.^50^^,^^51^ iNKT, γδT, and MAIT cells all share effector memory features and are highly sensitive to their environment and cytokine milieu. Moreover, innate-like T cells recognize non-polymorphic receptors, which are often upregulated in inflammatory contexts. Despite these common features, they recognize distinct molecular structures such as lipids, phospho-antigens, or microbial-derived metabolites that may account for the specificities of each subset we and others report in T1D and other autoimmune diseases.^50^^,^^51^

Overall, this study is primarily descriptive and based on peripheral blood, and therefore does not establish mechanistic causality. However, its main strength lies in providing a comprehensive and integrative analysis of innate-like T cells in adult T1D, addressing a previously fragmented and underexplored area. It fills a critical gap in the literature on iNKT and γδT cells in adult T1D, and provides a reference framework for future mechanistic studies. Beyond this descriptive value, our findings generate several testable hypotheses. The observed Th1 impairment in RO T1D suggests that early immune dysfunction may precede clinical onset, whereas the more pronounced exhaustion-like phenotype in long-term disease is consistent with chronic immune stress. Together, these observations support stage-associated immune alterations and pave the way for longitudinal and interventional studies. These studies should focus on the relationship between innate-like T cell phenotypes and variations in cytokines and microbial ligands able to activate these cells in patients with T1D.

### Limitations of the study

We recognize several limitations to this study. First, the cross-sectional design precludes any inference of causality or temporal evolution. Accordingly, the observed differences between RO and LT T1D should be interpreted as associations, generating hypotheses for future longitudinal studies. Second, the analysis was restricted to peripheral blood and may not fully reflect tissue-specific immune dynamics. Third, the number of patients with RO T1D was relatively small, particularly for analyses involving intracellular markers, which have limited statistical power. A larger sample size might enable the identification of additional marker correlations and further strengthen the observed differences between groups. In addition, Human Leukocyte Antigen (HLA) typing was not available for our cohort. Given the major role of HLA class I and II molecules in T1D susceptibility, immune heterogeneity and T cell activation, it is possible that some of the observed innate-like T cell alterations, such as cytokine production or activation and exhaustion-associated markers may vary according to HLA background. Furthermore, the predictive models were developed in a limited cohort and were not validated in an independent dataset. Although they achieved moderate discriminatory performance, the wide confidence intervals and modest sensitivity values indicate that these analyses should be considered exploratory and require validation in larger independent cohorts. Finally, correction for multiple comparisons, accounting for the total number of statistical tests, was not performed, as the groups and statistical analyses were pre-planned, as for the MAIT cell analysis.^15^ However, our conclusions are supported by several concordant parameters related to the phenotype and function of innate-like T cells.

## Resource availability

### Lead contact

Requests for further information and resources should be directed to and will be fulfilled by the lead contact, Dr. Agnès Lehuen, agnes.lehuen@inserm.fr.

### Materials availability

This study did not generate new unique reagents.

### Data and code availability

• The data supporting the findings of this study are available from lead contact upon reasonable request.
• All custom code used for data analysis has been deposited in GitHub (https://github.com/nelisa86/DiabeteNel) and archived on Zenodo (https://doi.org/10.5281/zenodo.21489688).
• Any additional information required to reanalyze the data reported in this paper is available from the lead contact upon request.

## Acknowledgments

We thank all the patients and the medical staff of Cochin Hospital, particularly Dr. A. Sola (Diabetology Department, Cochin Hospital, AP-HP Centre, France) and the Cybio Facilities of Institut Cochin. The graphical abstract was created using Biorender. This study was supported by grants from 10.13039/501100001677Inserm, 10.13039/501100004794CNRS; Laboratoire d'Excellence consortium Inflamex (grant no. ANR-18-IDEX-0001); ANR grants (ANR-17-CE14-0002-01, ANR-19-CE14-0041-01, ANR-20-CE14-0044, and ANR-24-CE15-7152); the 10.13039/501100002915Fondation pour la Recherche Médicale (10.13039/501100002915FRM grant no. EQU201903007779 to A.L. and FDT202204015005 to L. Bertrand); la Fondation Francophone pour la Recherche sur le Diabète to A.L.; and Aide aux Jeunes Diabétiques fellowships to I.N., G.V., and L. Bertrand.

## Author contributions

I.N. and G.V., conceptualization, data curation, formal analysis, investigation, writing – original draft, and writing – review and editing; L. Bertrand, formal analysis and writing – review and editing; L. Beaudoin, data curation and formal analysis; E.L., project administration, conceptualization, and resources; I.T., methodology, formal analysis, and writing – review and editing; A.L., conceptualization, methodology, supervision, project administration, formal analysis, validation, and writing – review and editing.

## Declaration of interests

The authors declare no competing interests.

## STAR★Methods

### Key resources table

**Table:** 

REAGENT or RESOURCE	SOURCE	IDENTIFIER
**Antibodies**		

Brilliant Violet 785™ mouse anti-human CD3 Antibody	BioLegend	OKT3; Cat#317329; RRID:AB_11219196
Brilliant Violet 711™ mouse anti-human CD4 Antibody	BioLegend	OKT4; Cat#317440; RRID:AB_2562912
Brilliant Violet 421™ mouse anti-human TCR Vα7.2 Antibody	BioLegend	3C10; Cat#351715; RRID:AB_2562534
PerCP/Cyanine5.5 mouse anti-human TCR Vα24-Jα18	BioLegend	6B11; Cat#342914; RRID:AB_2562455
Brilliant Violet 605™ mouse anti-human CD161 Antibody	BioLegend	HP-3G10; Cat#339915; RRID:AB_11142679
PE mouse anti-human CD196 (CCR6) Antibody	BioLegend	G034E3; Cat#353410; RRID:AB_10913815
Brilliant Violet 510™ mouse anti-human CD56 (NCAM) Antibody	BioLegend	HCD56; Cat#318340; RRID:AB_2561944
Brilliant Violet 650™ mouse anti-human CD69 Antibody	BioLegend	FN50; Cat#310933; RRID:AB_256178
Brilliant Violet 510™ mouse anti-human CD27 Antibody	BioLegend	O323; Cat#302835; RRID:AB_2561382
APC-Cy™7 mouse anti-human CD8	BD Biosciences	SK1; Cat#557834; RRID:AB_396892
FITC mouse anti-human PD1/CD279	BD Biosciences	MIH4; Cat#557860; RRID:AB_2159176
PE-Cy™7 mouse anti-human CD25	BD Biosciences	M-A251; Cat#557741; RRID:AB_396847
PE-CF594 mouse anti-human TCR γδ	BD Biosciences	B1; Cat# 562511; RRID:AB_2737631
Alexa Fluor™488 mouse anti-human BCL2	BioLegend	Clone 100; Cat#658704; RRID:AB_2563152
PE mouse anti-human Ki-67	BD Biosciences	B56; Cat#556027; RRID:AB_2266296
FITC mouse anti-human IFN-γ	BioLegend	4S B3; Cat#506504; RRID:AB_315437
PE mouse anti-human IL-2	BioLegend	MQ1-17H12; Cat#500306; RRID:AB_31509
Brilliant Violet 711™ mouse anti-human IL-17	BioLegend	BL168; Cat#512327; RRID:AB_11219603
Brilliant Violet 650™ mouse anti-human TNF-α	BioLegend	Mab11; Cat#502937; RRID:AB_2561355
APC mouse anti-human IL-10	BD Biosciences	JES3-19F1; Cat#554707; RRID:AB_398582
Brilliant Violet 510™ mouse anti-human Granzyme B	BD Biosciences	GB11; Cat#563388; RRID:AB_2738174
PE-Cy™7 mouse anti-human IL-4	BD Biosciences	8D4-8; Cat#560672; RRID:AB_1727547

**Biological samples**		

Peripheral blood mononuclear cells	Isolated from patients (Nel et al.^15^)	https://doi.org/10.1007/s00125-021-05527-y
Peripheral blood mononuclear cells	Isolated from one blood bag from a healthy donor (staining reference)	N/A

**Chemicals, peptides, and recombinant proteins**		

Phorbol 12-Myristate 13-Acetate (PMA)	Merck Sigma-Aldrich	Cat#P8139-5 MG
Ionomycin calcium salt	Merck Sigma-Aldrich	Cat#I0634-1 MG
Brefeldin A	Merck Sigma-Aldrich	Cat#B7651-5 MG

**Critical commercial assays**		

BD Cytofix/Cytoperm™ Fixation/Permeabilization Kit	BD Biosciences	Cat#554714; RRID:AB_2869008
eBioscience™ Intracellular Fixation & Permeabilization Buffer Set	eBioscience, Thermo Fisher Scientific	Cat#88-8824-00

**Deposited data**		

Custom R scripts for data analysis	Github, Zenodo	https://github.com/nelisa86/DiabeteNel, Zenodo: https://doi.org/10.5281/zenodo.21489688.
MAIT cell flow cytometry data from patients.	Nel et al.^15^	https://doi.org/10.1007/s00125-021-05527-y

**Software and algorithms**		

Excel 2016	Microsoft Corporation	https://www.microsoft.com/
FlowJo V10.1	BD Biosciences	https://www.flowjo.com/
GraphPad Prism version 5.00.288	GraphPad Software Inc.	https://www.graphpad.com/
R V4.2.3	The R Foundation	https://www.r-project.org/

### Experimental model and study participant details

Flow cytometry analysis of iNKT and γδT cells was performed on fresh peripheral blood samples from adult patients with T1D or healthy donors, patients which were also used to study MAIT cell alterations as previously described (Table S1).^15^ Ancestry, race, ethnicity, and socioeconomic status of participants were not considered in this study. This cross-sectional study included fresh blood samples collected from 55 healthy donors, 47 adults with long-term (LT) T1D (disease duration >2 years) and 21 adults with recent-onset (RO) T1D (within 10 days after diagnosis and insulin therapy initiation). All stainings and acquisitions were performed within 24 hours of blood collection to minimize variability due to sample degradation. Patients from all three groups (healthy donors, HD, recent-onset T1D, RO; and long-term T1D, LT) were recruited and processed in parallel within the same time period and according to onset, follow-up of the disease and availability of age-matched healthy donors at the French Blood Organisation (Etablissement Français du Sang, EFS). Participants with long-term T1D were matched to healthy donors for age, BMI and sex ratio. Importantly, no RO T1D patients included in this study presented acute metabolic complication at time of blood collection, nor did LT T1D patients present severe renal, cardiac or ophthalmological complications, or other active autoimmune diseases. All flow cytometry analyses were performed and analyzed blindly. Positivity thresholds were determined using Fluorescence Minus One (FMO) controls for all surface markers and cytokines.

Type 1 diabetes diagnosis was established according to current clinical guidelines, based on clinical presentation and biological criteria, and confirmed by the presence of at least one diabetes-associated autoantibody (GAD, IA-2, or ZnT8) at diagnosis.^15^ Individuals with latent autoimmune diabetes in adults (LADA), ketosis-prone diabetes, or ongoing infectious, inflammatory, autoimmune comorbidities, antibiotic or immunomodulatory treatments were also excluded from this study. Most samples were collected prior to the COVID-19 outbreak in France (February 2020); otherwise, inclusion required the absence of clinical symptoms and a negative SARS-CoV-2 PCR test. Our study was approved by the Ethics Committee (Comité de protection des personnes (CPP) Ile-de-France) (N°Eudra CT/ID-RCB: 2014-A00517-40) and all subjects provided written informed consent before blood donation.

### Method details

#### PBMC flow cytometry analysis

Detailed protocols for peripheral blood mononucleated cells (PBMCs) isolation and staining were previously described.^15^ PBMCs were isolated from fresh blood samples using Ficoll-Paque (Leucosep) tubes and then labeled at 4°C, protected from light, in PBS supplemented with 5%FCS, 0.1% sodium azide with the following anti-human monoclonal antibodies (mAbs): anti-CD3 (OKT3, BV785), CD4 (OKT4, BV711), Vα7.2 (3C10, BV421), Vα24Jα18 (6B11, PerCP-Cy5.5), D161 (HP-3G10, BV605), CCR6 (G034E3, PE), CD56 (HCD56, BV510), CD69 (FN50, BV650), CD27 (O323, BV510) from BioLegend; anti-CD8 (SK1, APC-Cy7), PD-1 (MIH4, FITC), CD25 (M-A251, PE-Cy7), anti-TCRγδ (B1, PE-CF594) from BD Biosciences and anti-CD127 (R34.34, PE-Cy5) from Beckman Coulter.

After surface staining, PBMCs were fixated and permeabilized using fixation-permeabilization buffer (eBioscience) and incubated at 4°C, protected from light, in perm-wash buffer (eBioscience) with anti-Ki67 (B56, PE) from BD Biosciences and anti-BCL-2 (clone 100, AF488) from BioLegend. For cytokines and granzyme B (GzB) detection, PBMCs were activated with phorbol myristate acetate (PMA) (25 ng/mL, Sigma-Aldrich) and ionomycin (1 μg/mL, Sigma-Aldrich) in the presence of brefeldin A (10 μg/mL, Sigma-Aldrich) for 6 h to assess maximal, standardized cytokine production in a TCR-independent manner. While this does not reflect physiological antigen-specific activation, it does enable direct comparison of intrinsic functional capacity across all innate-like T cell subsets. Stimulated cells were next surface-stained as described above and intracellularly-stained with the following mAbs: anti-IFN-γ (4S B3, FITC), IL-2 (MQ1-17H12, PE), IL-17 (BL168, BV711) and TNF-α (Mab11, BV650) from BioLegend; anti-IL-4 (8D4-8, PE-Cy7), IL-10 (JES3-19F1, APC) and GzB (GB11, BV510) from BD Biosciences. Data acquisition was performed using a BD Biosciences LSR-Fortessa cytometer or a FACSAria III cytometer and results were analyzed with FlowJo analysis software V10.1 (BD Biosciences). Polyfunctional cells were defined as cells co-expressing ≥3 cytokines following PMA/ionomycin stimulation.

Variation in sample size across panels reflects the experimental workflow and cell availability constraints. For each patient, surface marker staining was performed as a priority, while intracellular cytokine and Ki67/BCL-2 staining were performed depending on the number of remaining viable cells. Patients with fewer than 20 analyzable iNKT cells were excluded from the corresponding iNKT analyses.

Staining procedures and flow cytometer stability were verified by performing complete staining every 15 days on frozen aliquoted reference PBMCs, obtained from a blood bag provided by the EFS from a healthy adult donor, to ensure no fluctuation in staining intensity for surface markers, no drift in population frequencies and no variability in cytokine detection after PMA/ionomycin stimulation.

#### Multivariate analyses

Multiparameter analysis pie charts were created using Excel 2016 and the Boolean gating option of Flowjo software V10.1 (Tree Star). Correlograms were generated with the corrplot^52^ and Hmisc^53^ packages. Principal Component Analysis (PCA) was performed using the FactoMineR^54^ and Factoextra packages.^55^

#### Prediction models

Prediction models were applied to assess diagnostic accuracy based on the available immunological data and to identify the variables contributing most strongly to classification performance. Prediction models were generated using the randomForest^56^ and earth^57^ packages. Model training and hyperparameter tuning were conducted using the caret package in R. For the MARS model, tuning was performed using a predefined grid with the number of pruned terms (nprune) ranging from 2 to 10 and interaction degree from 1 to 3. Tuning was conducted on a randomly selected subset comprising 70% of the patient cohort. For the random forest model, hyperparameters were tuned by varying the number of variables randomly sampled at each split (mtry) from 5 to 20 and the minimum node size from 1 to 10. Both models were trained using repeated cross validation, consisting of 10-fold cross validation repeated five times, to obtain robust performance estimates and reduce variability due to random data partitioning. Predictor variables were centered and scaled prior to model fitting to prevent variables with larger numerical ranges from disproportionately influencing variable importance measures. During training, class probabilities were estimated to enable probabilistic performance assessment. Model optimization was guided by the area under the receiver operating characteristic curve (AUC), using the twoClassSummary function as the tuning metric. Predictions from the final tuned models were retained for downstream analyses. Receiver operating characteristic (ROC) curves for the best performing models were constructed on the independent test set using the pROC package. Variable importance was further assessed using permutation-based importance estimation with 50 Monte Carlo simulations. Importance scores were reported as the mean decrease in AUC, with variability reflecting permutation induced instability, as computed using the vip package in R. Regarding class balance, the LT restricted analysis included balanced groups (*n* = 34 LT patients and *n* = 34 healthy controls), and no class balancing procedure was therefore required. In the combined LT and RO analysis, a mild class imbalance was present (*n* = 44 diabetes patients vs. *n* = 34 healthy controls). Given the moderate nature of this imbalance, no additional resampling procedure was applied. Model optimization was guided by the AUC, a metric that is robust to mild class imbalance compared to accuracy-based measures.

Missing values were handled by retaining only complete observations for PCA and predictive modeling analyses. This filtering resulted in the exclusion of 11 RO patients (from *n* = 21 to *n* = 10), 13 LT patients (from *n* = 47 to *n* = 34), and 21 controls (from *n* = 55 to *n* = 34), yielding a final analytical cohort of 78 participants (*n* = 10 RO, *n* = 34 LT, and *n* = 34 controls). No imputation method was applied.

### Quantification and statistical analysis

All analyses were conducted using GraphPad Prism version 5.00.288 (GraphPad Software Inc., San Diego, CA, United States) or the R software V4.2.3. Statistical parameters are indicated in figure legends. Datasets were compared using either non-parametric tests two-tailed Wilcoxon-Mann-Whitney test or Spearman’s correlation test. Primary group comparisons (HD vs. RO and HD vs. LT) were pre-planned and hypothesis-driven. Given the exploratory nature of several secondary analyses, including correlation analyses and multiparametric profiling, correction for multiple comparisons was thus not applied across all tests. Therefore, *p* values should be interpreted cautiously, particularly for exploratory analyses, and emphasis was placed on consistent biological patterns observed across multiple related parameters. To test whether the groups (healthy donors, recent-onset T1D patients, and long-term T1D patients) were significantly distinct in the multivariate (PCA) space, we performed a permutational multivariate analysis of variance (PERMANOVA) using the *adonis2* function from the vegan package (v2.x) in R. Euclidean distance was used as the dissimilarity measure, and the test was performed with 999 permutations. Results were considered statistically significant for *p*-values <0.05 (∗, *p* < 0.05; ∗∗, *p* < 0.01, ∗∗∗, *p* < 0.001).
