# Supplementary material for: Similarities and singularities between innate-like T cell subsets in adults with type 1 diabetes
Source: iScience. 2026 Jul 27;29(8):116957. doi: 10.1016/j.isci.2026.116957 (PMC13446199; doi:10.1016/j.isci.2026.116957)
Supplement: Supplementary file 1 [file mmc1.pdf]

iScience, Volume *29*

Supplemental information

Similarities and singularities between innate-like T cell subsets in adults with type 1 diabetes

Isabelle Nel, Gaëlle Vermillac, Léo Bertrand, Lucie Beaudoin, Etienne Larger, Ioannis Theodorou, and Agnès Lehuen

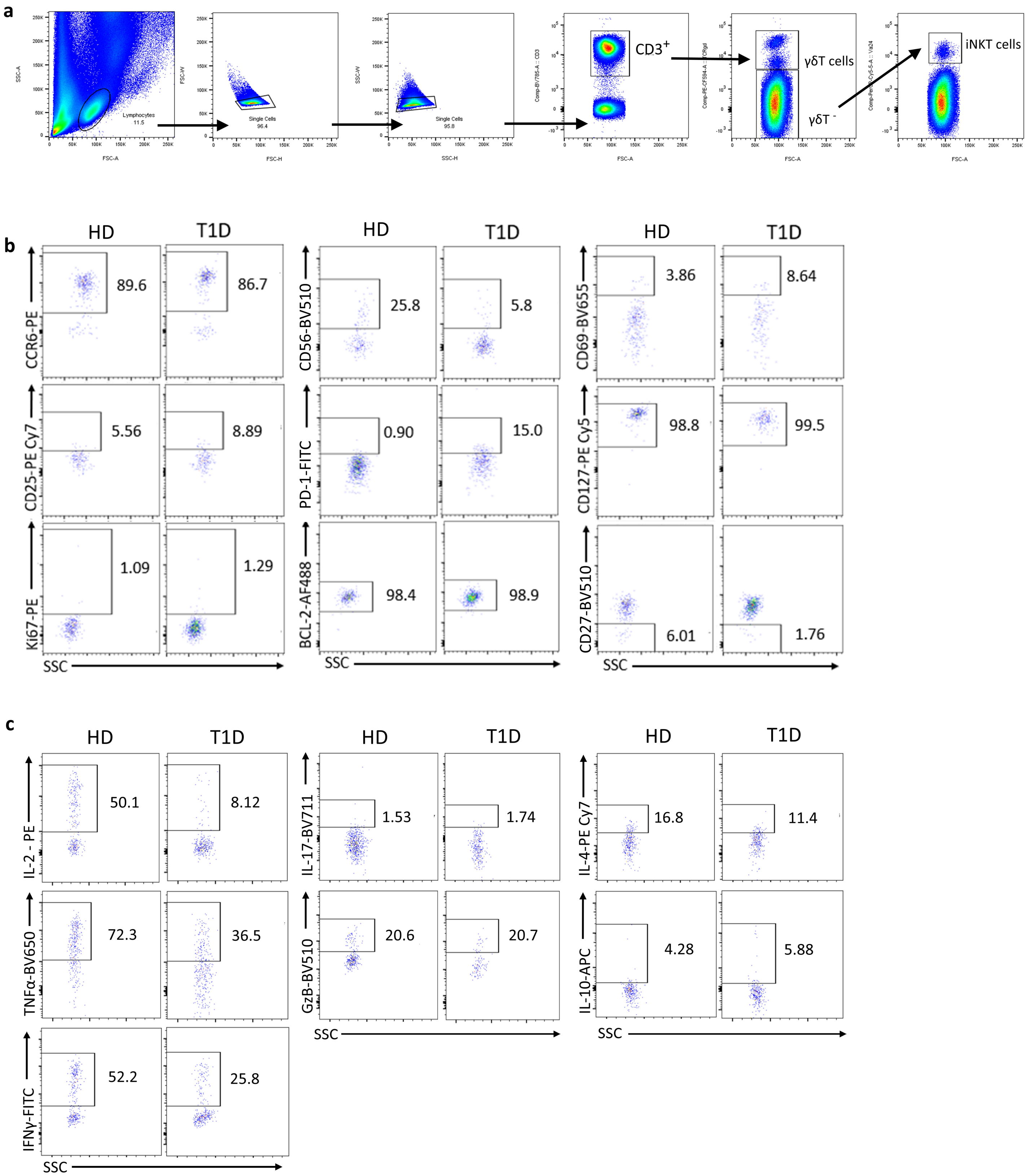

Figure S1. Flow cytometry gating strategy for iNKT, γδT cell identification, and iNKT cell activation, exhaustion and function. Related to Fig.1-3. **(a)** Surface gating strategy, in the blood of a healthy donor, of circulating iNKT and γδT cell frequencies. **(b)** Representative dot plot of CCR6$^+$, CD56$^+$, CD69$^+$, CD25$^+$, PD1$^+$, CD127$^+$, Ki67$^+$, BCL-2$^+$, and CD27^{neg} iNKT cells in blood from a healthy adult donor (HD) and an adult patient with type 1 diabetes (T1D). **(c)** Representative dot plot of IL-2$^+$, IL-17$^+$, IL-4$^+$, TNF-α$^+$, granzyme B (GzB)$^+$, IL-10$^+$ and IFN-γ$^+$ iNKT cells in blood from a healthy adult donor (HD) and an adult patient with type 1 diabetes (T1D). Percentage cell frequencies are shown next to the outlined areas. FSC, Forwardscatter. SSC, Sidescatter.

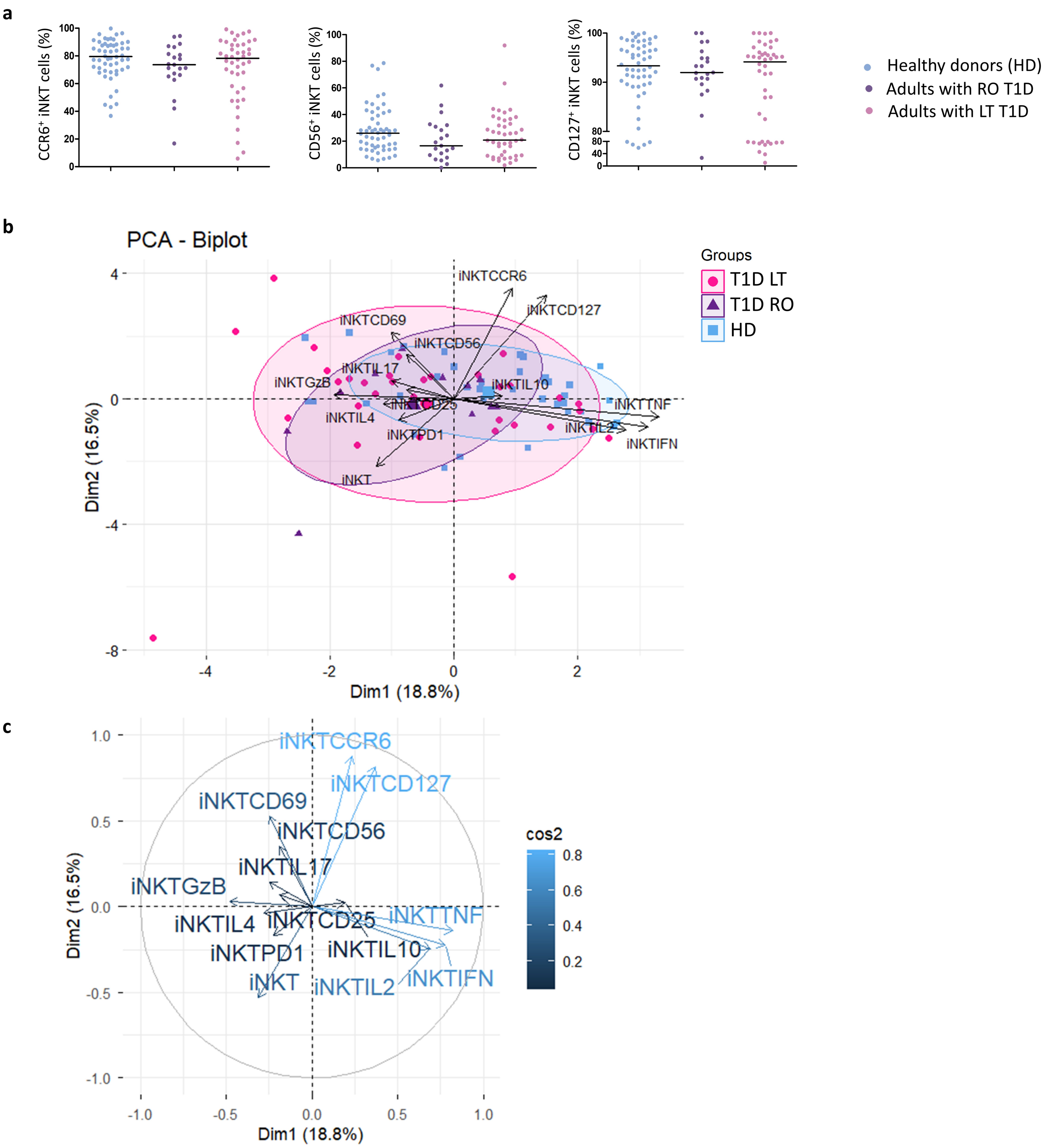

Figure S2. iNKT cell cytokine production is associated to disease status in patients with type 1 diabetes. Related to Fig. 1-2. (a) Flow cytometry analysis of blood CCR6⁺, CD56⁺ and CD127⁺ iNKT cells in healthy donors (HD, n=54), patients with recent-onset (RO, n=21) or long-term (LT, n=44) type 1 diabetes (T1D). **(b, c)** Principal Component Analysis (PCA) of healthy donors (HD, n=40) recent-onset (RO, n=11) and long-term (LT, n=37) adult patients with type 1 diabetes, using iNKT cells frequency, phenotype and function markers as variables. Biplot PCA projection represents the first and second dimensions. **(b, c)** Arrows represent the contributions of each quantitative variable and concentration ellipses indicate 95% confidence intervals for each group (b). **(c)** Arrows are coloured based on the square cosine, which is representative of variable fit on the dimensions. Each symbol represents an individual subject. Small horizontal lines indicate the median with the interquartile range. No significative differences were found between datasets (non-parametric two-tailed Mann-Whitney test (b)).

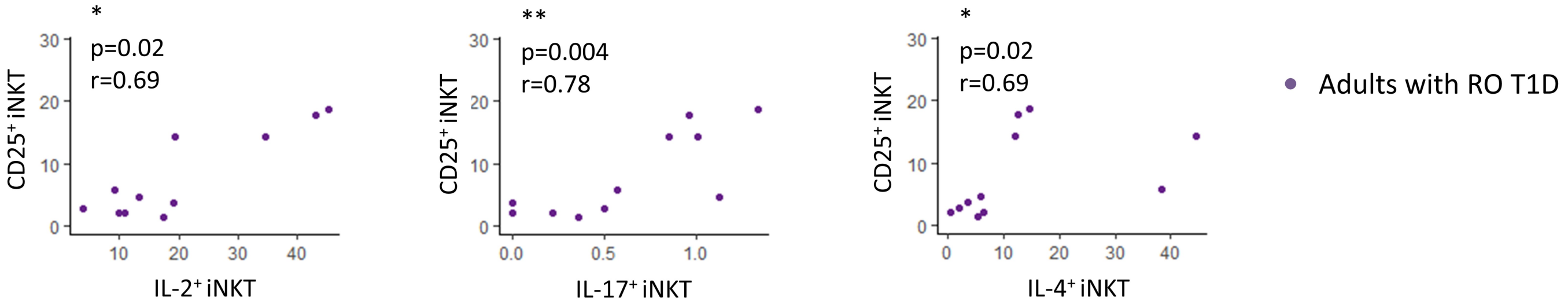

Figure S3. iNKT cell cytokine production correlates with activation in patients with recent-onset type 1 diabetes. Related to Fig. 2. Correlations between $CD25^+$ iNKT cell frequency and IL-2^+, IL-17^+ and IL-4^+ iNKT cell frequency in adult patients with recent-onset (RO) type 1 diabetes (T1D) (n=11). Each symbol represents an individual subject. Small horizontal lines indicate the median with the interquartile range. * $p < 0.05$, ** $p < 0.01$ (Spearman's correlation test).

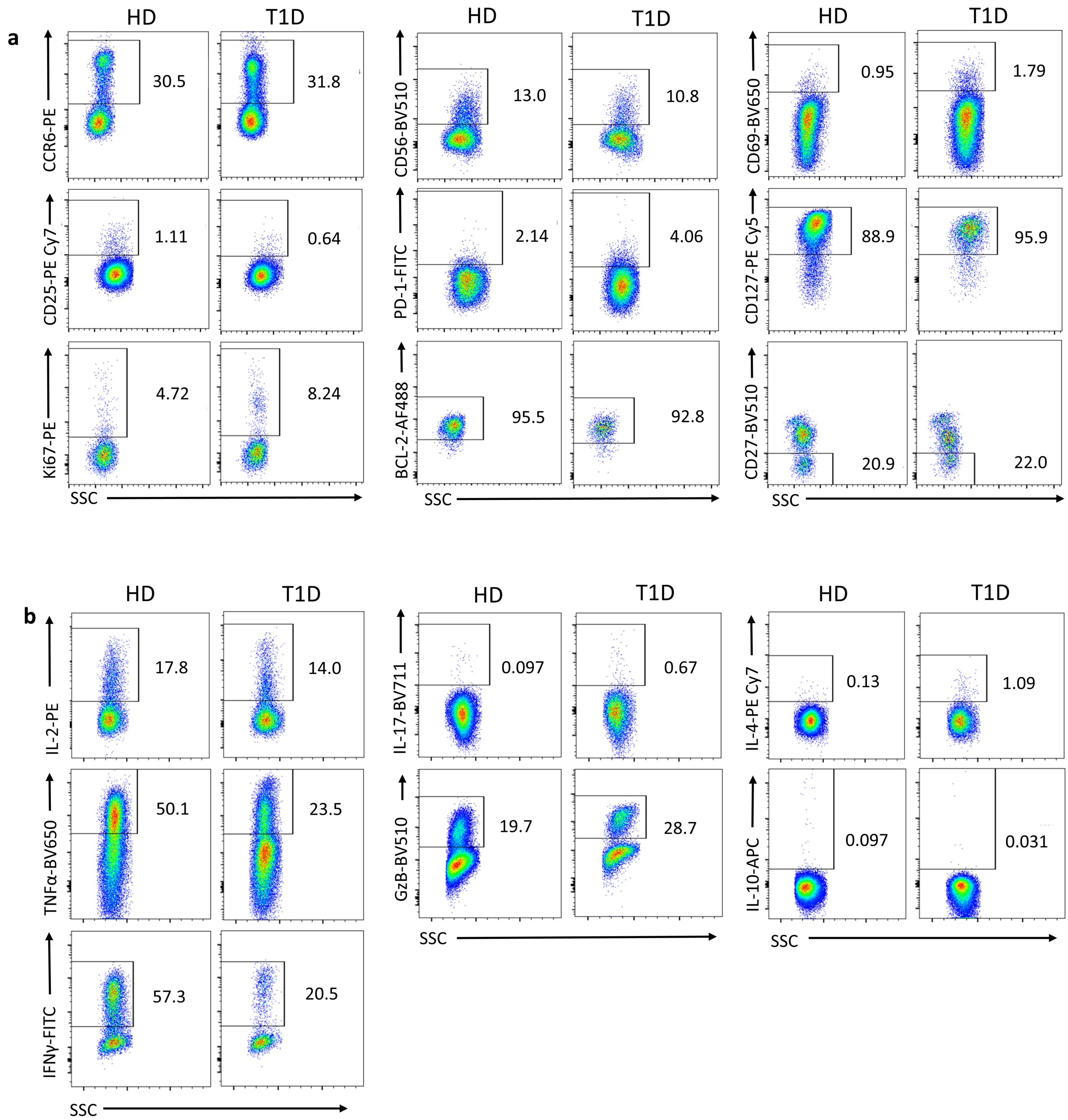

Figure S4. Flow cytometry gating strategy for γδT cell activation, exhaustion and function. Related to Fig. 3-4. **(a)** Representative dot plot of CCR6$^+$, CD56$^+$, CD69$^+$, CD25$^+$, PD1$^+$, CD127$^+$, Ki67$^+$, BCL-2$^+$, and CD27^{neg} γδT cells in blood from a healthy adult donor (HD) and an adult patient with type 1 diabetes (T1D). **(b)** Representative dot plot of IL-2$^+$, IL-17$^+$, IL-4$^+$, TNF-α$^+$, granzyme B (GzB)$^+$, IL-10$^+$ and IFN-γ$^+$ γδT cells in blood from a healthy adult donor (HD) and an adult patient with type 1 diabetes (T1D). Percentage cell frequencies are shown next to the outlined areas. SSC, Sidescatter.

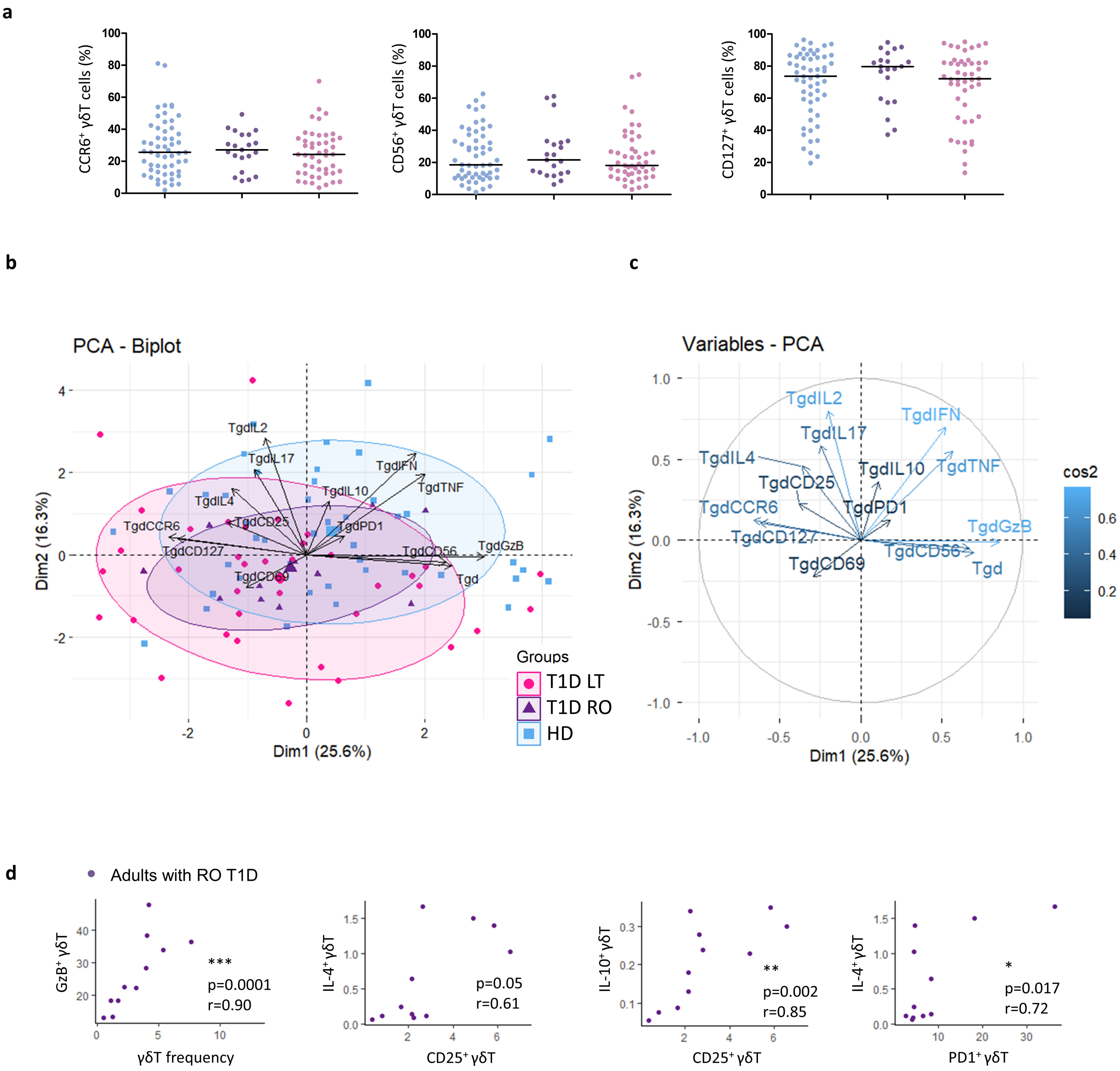

Figure S5. γδT cell cytokine production is associated to activation and disease status in patients with type 1 diabetes. Related to Fig. 3-4. (a) Flow cytometry analysis of blood CCR6⁺, CD56⁺ and CD127⁺ γδT cells in healthy donors (HD, n=55), patients with recent-onset (RO, n=21) or long-term (LT, n=47) type 1 diabetes (T1D). **(b, c)** Principal Component Analysis (PCA) of healthy donors (HD, n=45) recent-onset (RO, n=11) and long-term (LT, n=38) adult patients with type 1 diabetes, using γδT cells frequency, phenotype and function markers as variables. Biplot PCA projection represents the first and second dimensions. **(b, c)** Arrows represent the contributions of each quantitative variable and concentration ellipses indicate 95% confidence intervals for each group **(b)**. **(c)** Arrows are coloured based on the square cosine, which is representative of variable fit on the dimensions. **(d)** Correlations between granzyme B (GzB)⁺ γδT cell and total γδT cell frequency, IL-4⁺ γδT cell and CD25⁺ γδT cell frequencies, IL-10⁺ γδT and CD25⁺ γδT cell frequencies, IL-4⁺ γδT cell and PD1⁺ γδT cell frequencies in patients with recent-onset (RO, n=11) type 1 diabetes (T1D). Each symbol represents an individual subject. Small horizontal lines indicate the median with the interquartile range. * p <0.05, ** p <0.01, *** p <0.001 (non-parametric two-tailed Mann-Whitney test **(a)** or Spearman's correlation test **(d)**).

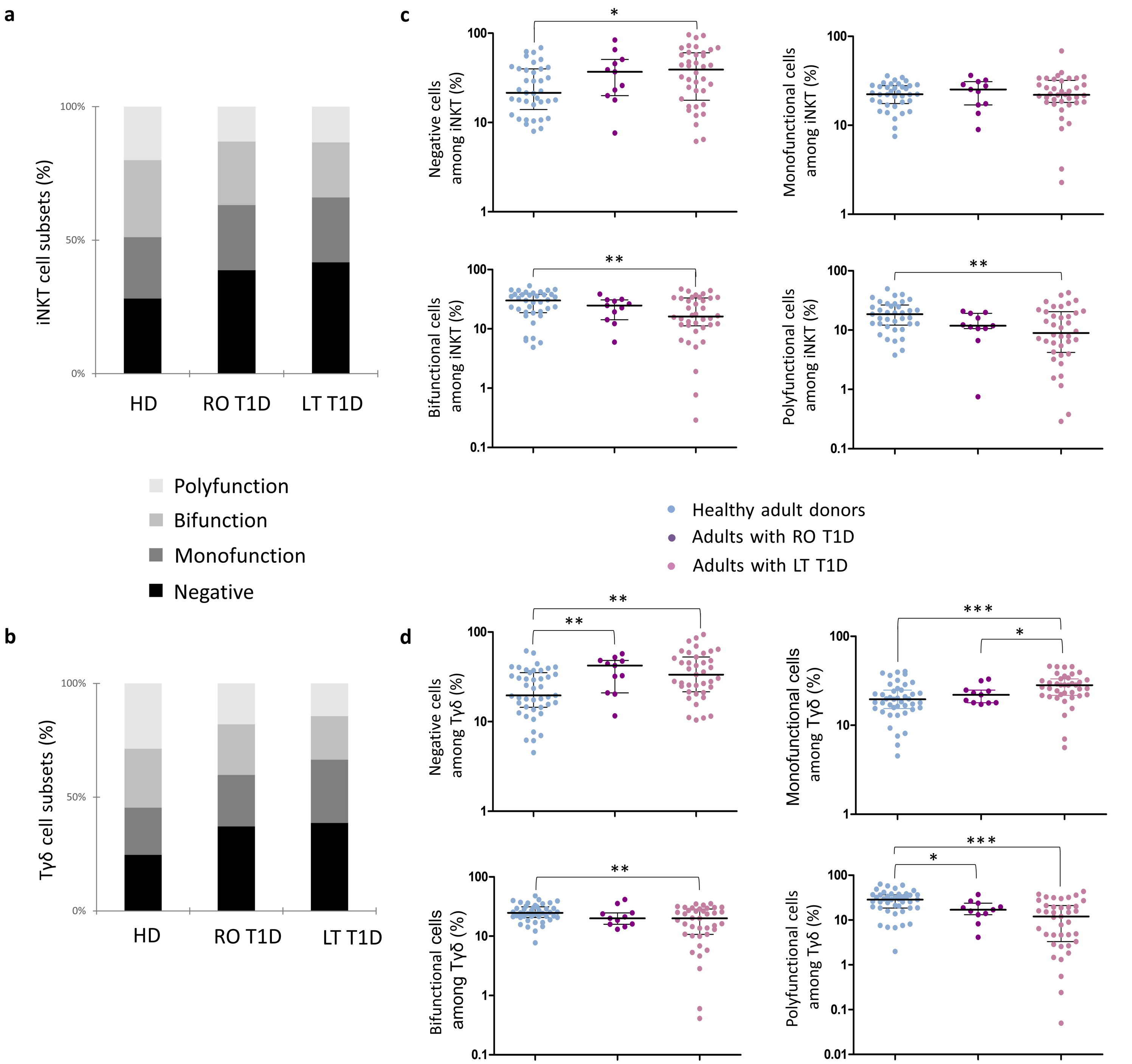

Figure S6. Polyfunctional alterations of circulating iNKT and γδT cells in adults with type 1 diabetes. Related to Fig. 5. Bar plot representative of cumulative Th1 cytokines, IL-17 and GzB production by iNKT cells **(a)** or γδT cells **(b)**, classified as producing none (negative), one (monofunction), two (bifunction) or more than two (polyfunction) of these factors. **(c)** Flow cytometry analysis of negative, monofunctional, bifunctional and polyfunctional iNKT cells as described in **(a)** in healthy donors (HD, n=37), adults with recent-onset (RO, n=11) or long-term (LT, n=38) type 1 diabetes (T1D). **(d)** Flow cytometry analysis of negative, monofunctional, bifunctional and polyfunctional γδT cells as described in **(b)** in healthy donors (HD, n=43), adults with recent-onset (RO, n=11) or long-term (LT, n=39) type 1 diabetes (T1D). Small horizontal lines indicate median with interquartile range. * $p < 0.05$, ** $p < 0.01$, *** $p < 0.001$ (non-parametric two-tailed Mann-Whitney test).

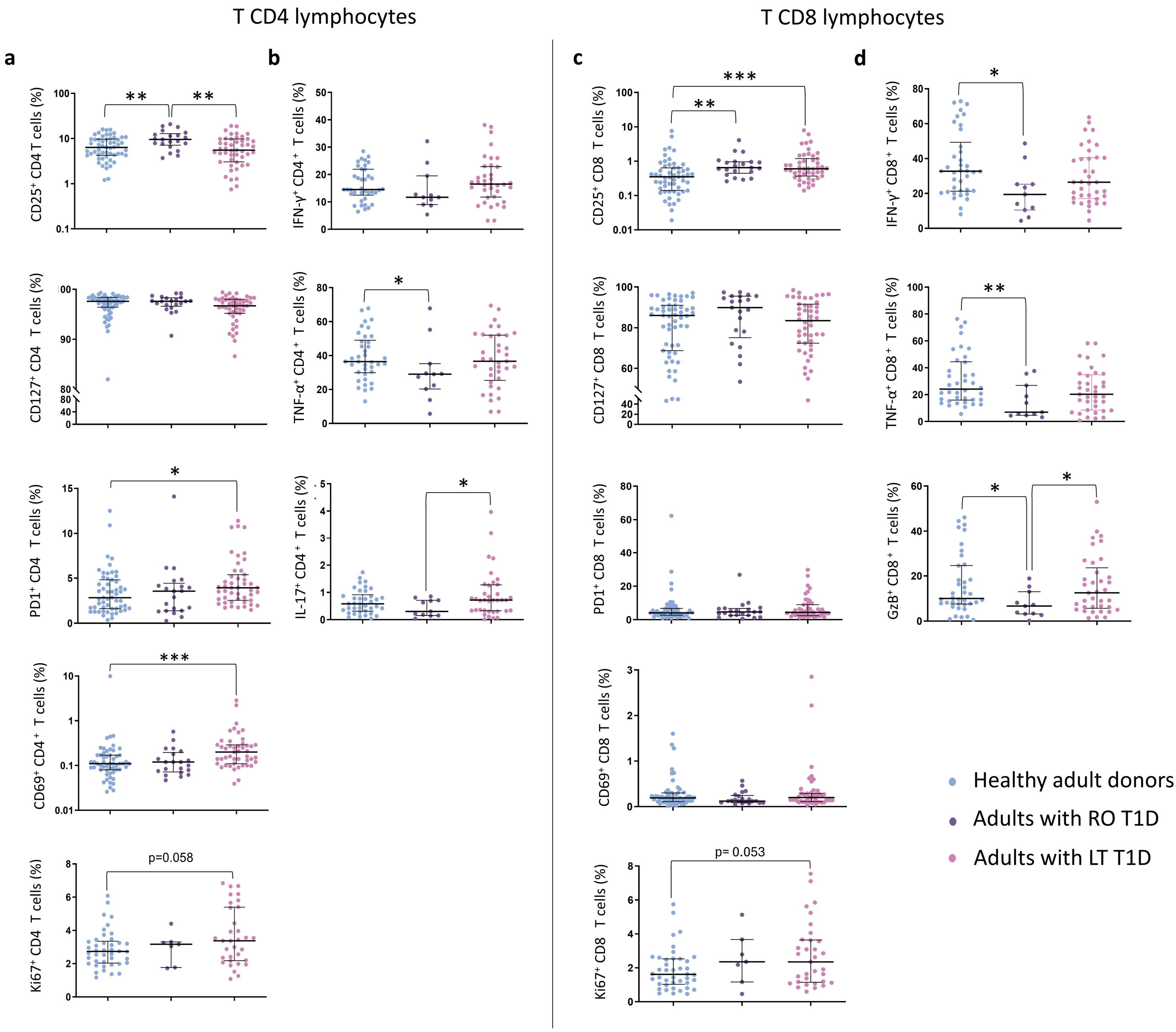

Figure S7. Alterations of conventional CD4 and CD8 T cells in patients with type 1 diabetes. Related to Fig. 6. (a) Flow cytometry analyses of blood CD25+, CD127+, PD1+, CD69+ and Ki67+ CD4 T cell frequencies in healthy donors (HD, n=42-55), recent-onset (RO, n=7-21) or long-term (LT, n=33-47) adult patients with type 1 diabetes (T1D). **(b)** Flow cytometry analyses of blood IFN-γ+, TNF-α+ and IL-17+ CD4 T cell frequencies in healthy donors (HD, n=38), recent-onset (RO, n=11) or long-term (LT, n=37) adult patients with type 1 diabetes (T1D). **(c)** Flow cytometry analyses of blood CD25+, CD127+, PD1+, CD69+ and Ki67+ CD8 T cell frequencies in healthy donors (HD, n=42-55), recent-onset (RO, n=7-21) or long-term (LT, n=33-47) adult patients with type 1 diabetes (T1D). **(d)** Flow cytometry analyses of blood IFN-γ+, TNF-α+ and granzyme B (GzB)+ CD8 T cell frequencies in healthy donors (HD, n=38), recent-onset (RO, n=11) or long-term (LT, n=37) adult patients with type 1 diabetes (T1D). Small horizontal lines indicate median with interquartile range. * p <0.05, ** p <0.01, *** p <0.001 (non-parametric two-tailed Mann-Whitney test).

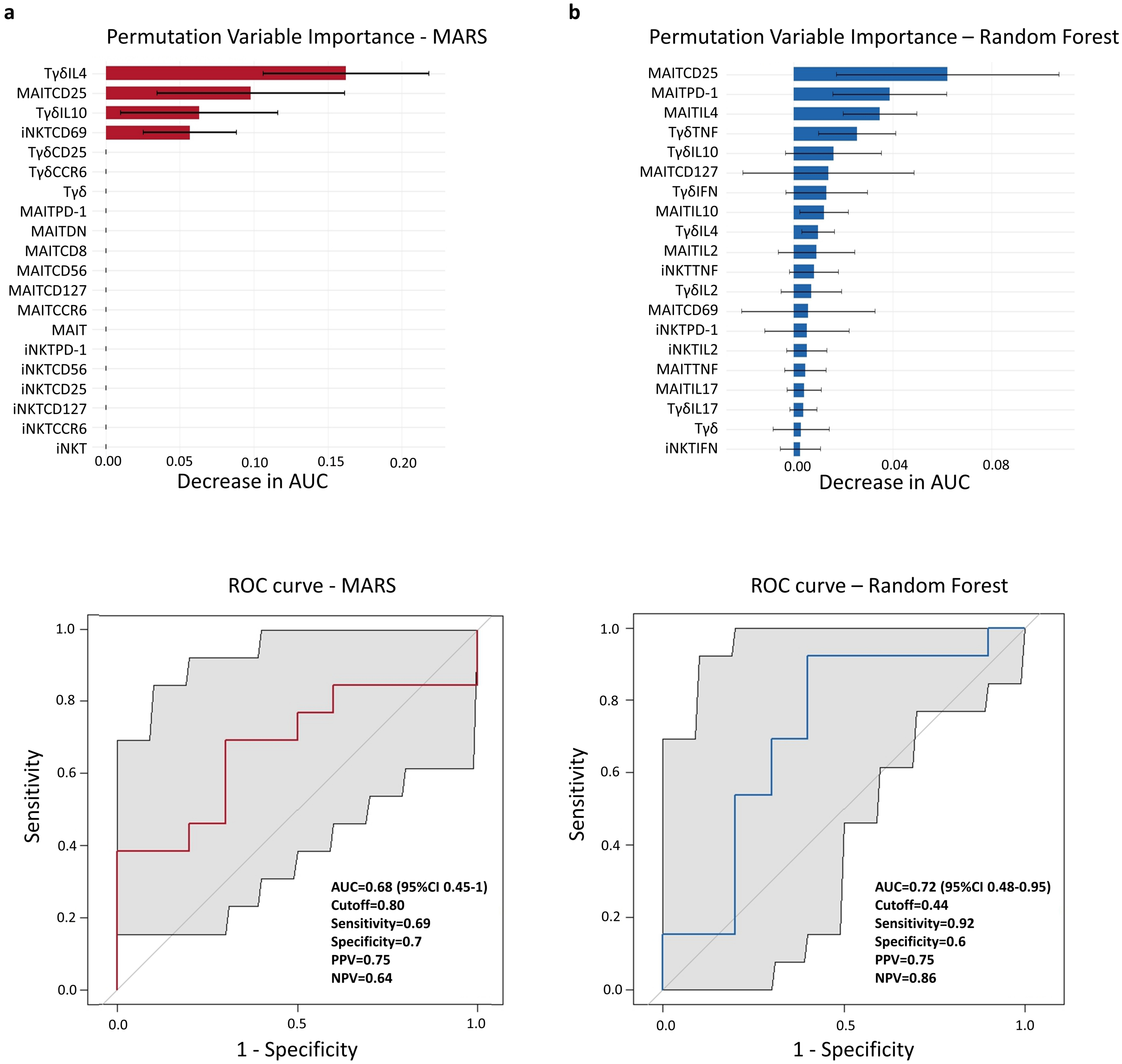

Figure S8. Exploratory analyses using predictive modelling according to the "diabetes" phenotype. Related to Fig. 6. MARS **(a)** and Random forest **(b)** predictive models output of major innate-like T cell variables that are discriminative for type 1 diabetes phenotype, resulting from the association of patients with recent-onset T1D and patients with long-term T1D. Input for each model included MAIT cell, iNKT cell, γδT cell frequency, phenotype and function markers as variables. For each predictive model, the upper panel shows the results of the variable permutation analysis, highlighting the most contributory variables. The lower panel corresponds to the associated ROC curve.

	Healthy Donors (n=55)	Recent onset T1D (n=21)	Long-term T1D (n=47)	p
**Age, years**	37.6±13.5 (22.2–67.3)	30.0±12.9 (16.9–69.1)	38.1±14.6 (19.8–74.1)	0.06
**BMI, kg/m2**	22.9±3.4 (18.0–32.6)	21.4±2.9 (16.6–27.7)	24.0±4.4 (17.4–39.6)	0.02 (*)
**Sex ratio, M/F**	25/30	11/10	20/27	0.75
**T1D duration**	–	6±4 days (2–14)	18.2±13.2 years (2–53)	< 0.001 (****)
**HbA1c, mmol/L**	–	97.1±29.0 (41.0–168.9)	63.0±18.2 (32.2–125.1)	< 0.001 (****)
**HbA1c, %**	–	11.0±2.7 (5.9–17.6)	7.9±1.7 (5.1–13.6)	< 0.001 (****)
**Fructosamine, µmol/L**	–	413.5±99.0 (248–612)	387.2±89.6 (248–612)	0.29
**Daily insulin dose, UI/kg**	–	0.6±0.3 (0.2–1.4)	0.6±0.2 (0.2–1.4)	> 0.9999
**Treatment regimens**<br>**Insulin pump therapy, n(%)**<br>**Multiple daily injection , n(%)**	-	13 (61.9)<br>8 (38.1)	20 (42.5)<br>27 (57.5)	0.15
**C peptide, nmol/L**		0.14±0.09 (0.04–0.4)	0.01±0.005 (0.01–0.03)	< 0.001 (****)
**Patients with complications , n(%)**<br>**Mild retinopathy, n(%)**<br>**Neuropathy, n(%)**<br>**Nephropathy, n(%)**	-	0 (0)	13 (27.6)<br>13 (27.6)<br>0 (0)<br>1 (2.1)	0.003
**Patients with associated MAI , n(%)**	-	0 (0)	1 (2.1)	0.31

Table S1. Clinical and biological characteristics of individuals with type 1 diabetes and healthy donors. Continuous variables are presented as mean ± SD (range) unless stated otherwise and compared using ANOVA or Welch's t-test as appropriate. Categorical variables were compared using the chi-square test or Fisher's exact test. A p-value < 0.05 was considered statistically significant.

F, female; M, male; T1D, type 1 diabetes.
